# World wide spatial capital

**DOI:** 10.1371/journal.pone.0190346

**Published:** 2018-02-08

**Authors:** Rijurekha Sen, Daniele Quercia

**Affiliations:** 1 Indian Institute of Technology Delhi, Delhi, India; 2 Nokia Bell Labs, Cambridge, United Kingdom; University of Oxford, UNITED KINGDOM

## Abstract

In its most basic form, the spatial capital of a neighborhood entails that most aspects of daily life are located close at hand. Urban planning researchers have widely recognized its importance, not least because it can be transformed in other forms of capital such as economical capital (e.g., house prices, retail sales) and social capital (e.g., neighborhood cohesion). Researchers have already studied spatial capital from official city data. Their work led to important planning decisions, yet it also relied on data that is costly to create and update, and produced metrics that are difficult to compare across cities. By contrast, we propose to measure spatial capital in cheap and standardized ways around the world. Hence the name of our project “World Wide Spatial Capital”. Our measures are cheap as they rely on the most basic information about a city that is currently available on the Web (i.e., which amenities are available and where). They are also standardized because they can be applied in any city in the five continents (as opposed to previous metrics that were mainly applied in USA and UK). We show that, upon these metrics, one could produce insights at the core of the urban planning discipline: which areas would benefit the most from urban interventions; how to inform planning depending on whether a city’s activity is mono- or poly-centric; how different cities fare against each other; and how spatial capital correlates with other urban characteristics such as mobility patterns and road network structure.

## 1 Introduction

The extent to which a neighborhood has most aspects of daily life located close at hand is a form of capital, and it is often called ‘spatial capital’. Any form of capital can be accumulated and transformed into other forms of capital. Spatial capital translates into economic capital, for example. This is evident from 5–10% increase in house prices in the United States, owing to better accessibility in residential neighborhoods [1, 2]. Easing access to daily necessities is also considered effective at both mitigating the obesity related health-care crisis in the USA [3] and reducing car dependency and fuel consumption, thereby enhancing environmental sustainability [4]. All this, in turn, makes a neighborhood significantly more livable, increasing its ‘social capital’, that is, the networks of relationships among residents that enable the neighborhood to function effectively [5].

As we will discuss in Section 5, researchers have studied spatial capital, but, collectively, they: i) ended up proposing different metrics for different cities; ii) relied on official and proprietary data that is costly to create and update; and iii) produced insights for selected cities, mainly in USA and UK, because of limited data availability.

To partly fix these three issues, we have launched a new project called “World Wide Spatial Capital”. The project’s site http://goodcitylife.org/spatialcapital makes data and research insights readily available. As part of this project, this article makes six main contributions:

1. Engineer a methodology to gather Google Places data at scale (Section 2.1). The resulting data is about twenty-five cities in five continents, including cities in the developing countries, which have received little attention in the urban literature so far.
2. Propose a spatial capital metric that can be computed from that place data (Section 2.2). The metric is cheap (it can be computed from Web data), standardized (it does not change across cities), and holds across the world.
3. Study how our metric is effective at determining desirable urban interventions in a city (Section 3.1). More specifically, we study whether we could determine which areas in a city would benefit the most from the least intervention. We show that it is indeed possible to identify areas with poor spatial capital and to recommend the introduction of new amenities.
4. Study how our metric is effective at determining centers of activity in a city (Section 3.2). We are able to measure a key concept in urban planning—poly-centricity. That is, whether a city’s activity is mono or poly-centric. We are able to offer insights for cities that have never been studied before (e.g., Rio-de-Janeiro and New Delhi).
5. Study how our metric is effective at comparing cities (Section 3.3). We are able to determine how cities fare against each other. We see that European neighborhoods enjoy higher spatial capital than what US ones do, as one expects [6, 7]. We also see that most cities in developing countries show poor access to spatial capital. The two exceptions are Singapore and Buenos Aires. Singapore, a first world city, has poor walkability in certain areas (likely because of zoning laws [8]). Buenos Aries, on the other hand, enjoys good walkability throughout the city (likely because of recent interventions [9]).
6. Study how our metric is correlated with other urban characteristics like diurnal and weekly mobility patterns and structural organization of the road network (Section 3.4), adding external validity to our study.

This is an application-oriented paper in the area of urban analytics that makes new contributions to both urban and web research. In urban research, this work offers a new methodology that exploits web data to tackle urban science challenges (e.g., mapping of spatial capital, poly-centricity, cross-city comparisons) at world scale for the first time. This work is also in line with recent web research in urban computing, which has analyzed large-scale urban dynamics [10–12]. There is a growing demand for making cities smarter, and web research (when properly informed by domain knowledge) could contribute with practical yet principled solutions.

## 2 Data and methods

We now describe the ways in which we have gathered and analyzed our datasets.

### 2.1 Crawling urban web data

To determine what is accessible from an area, it is necessary to measure the walking distances between the area and different amenities. We opt for the Google Maps public API mainly because it has the best coverage around the world (https://developers.google.com/maps/coverage).

This paper proposes a crawling methodology that is *reproducible* (others can repeat it) and *scalable* (the collection of data for a variety of cities does not require a prohibitive number of API calls). It consists of three main steps:

**Step 1**. A city is divided into 200*m* x 200*m* square grids. The size of 200*m* has been extensively used in prior work [13, 14] and is associated with the typical distance people are willing to walk daily (i.e., 400 meters). Each square is called area.
**Step 2**. An amenity category and the center of each square in the city (in the form of < *lat*, *lon* > point) are both given to the Google Places API (https://developers.google.com/places/). The result is a list of amenities in that category and their positions. In theory, this query needs to be repeated for all 50 categories. In practice, to minimize the number of API calls, similar categories are combined together using the ‘|’ (or) operator, and the query is repeated for 30 aggregate categories (Table 1).
**Step 3**. For each area, the closest amenities in each category is selected. We associate only one amenity with an area because we wish to capture only whether an amenity category is accessible or not within walking distance. To determine what is closest, we use the Google Distance Matrix API to get both distance and walking time (https://developers.google.com/maps/documentation/distancematrix/).

**Table 1:** List of amenity categories.

Categories	Purposes
bar—restaurant, bakery, cafe, convenience store—grocery or supermarket	food and daily necessities
bus station, taxi stand, train station—subway station, bicycle store, parking, gas station	transportation
shopping mall—department store, clothing store—shoe store—jewelry store	shopping and retail
doctor—dentist, hospital, beauty salon—hair care—spa—gym	health services
atm—bank	financial services
school—university	education
art gallery—museum, book store, library, movie rental, movie theater, night club	entertainment and tourism
stadium, amusement park—rv park—campground—zoo—aquarium, park	sports and outdoor activities
fire station, police	safety
church—hindu temple—mosque—place of worship—synagogue	religion

Those three steps are repeated for 25 cities in both developed and developing countries across the five continents (Table 2).

**Table 2:** Cities under study.

Cities	Characteristics
Barcelona, Berlin, London Milan, Paris, Rome	Industrialized; Europe.
Chicago, New York, San Francisco Seattle, Toronto, Washington	Industrialized; North America.
Beijing, Singapore, Tokyo	Industrialized; Asia.
Bengaluru, Buenos Aires, Delhi, Jakarta, Kuala Lampur, Mexico, Moscow, Mumbai, Nairobi, Rio	Developing; India, South America, Africa.

### 2.2 Defining the spatial capital metric

The crawled data is represented in the form of an area-by-category distance binary matrix *M*. The rows represent the areas in the city, which are the centers of the 200*m* X 200*m* squares, and the columns are the 30 categories described in Table 1. The value for each area-category cell is 1, if area *a* offers category *c* within *d* meters; otherwise, it is 0. Since the walking distance that people are typically willing to traverse on foot is 400 meters [15, 16], we present the results for *d* = 400. However, we produced the results for other distances, i.e., *d* = {200, 600, 800}, which all leads to similar conclusions. From the matrix’s rows, we compute area *a*’s spatial capital:

$$\begin{array}{c} C_{a}=\sum_{c}w_{c}*M_{ac} \end{array}$$ (1)

where *M* is our binary matrix, and *w*_*c*_ is a category weight that reflects the category’s importance. The importance changes depending on the issue at hand. For example, in a study of daily habits, the weight assigned to grocery stores is higher than that assigned to shoe stores. In this paper, for simplicity, we use *w*_*c*_ = 1 for all categories. In other studies, researchers could set the weights as they see fit.

### 2.3 Quality of Google Places data

**Fig 1:**
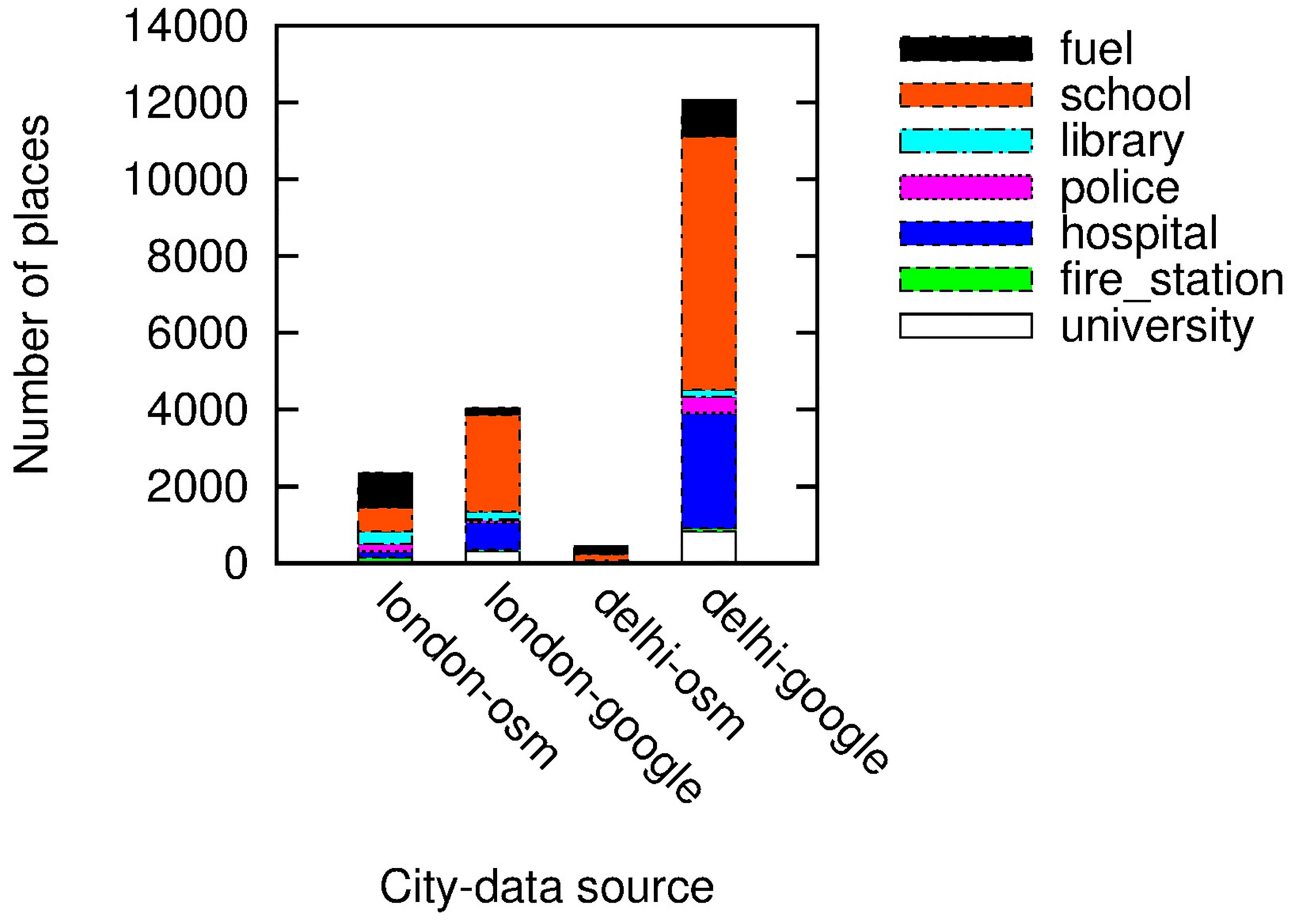
Open street map vs. Google.

**Fig 2:**
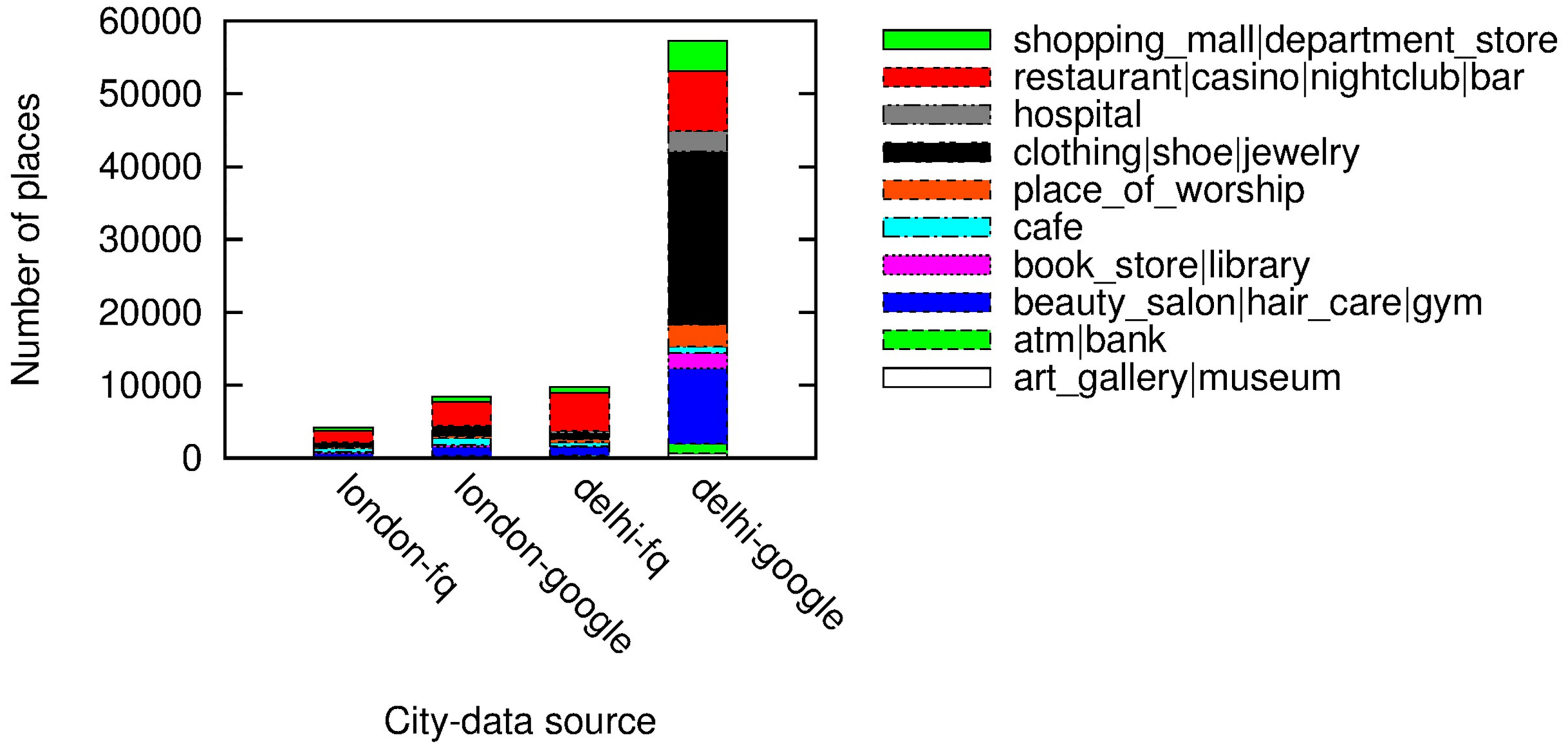
Foursquare vs. Google.

**Fig 3:**
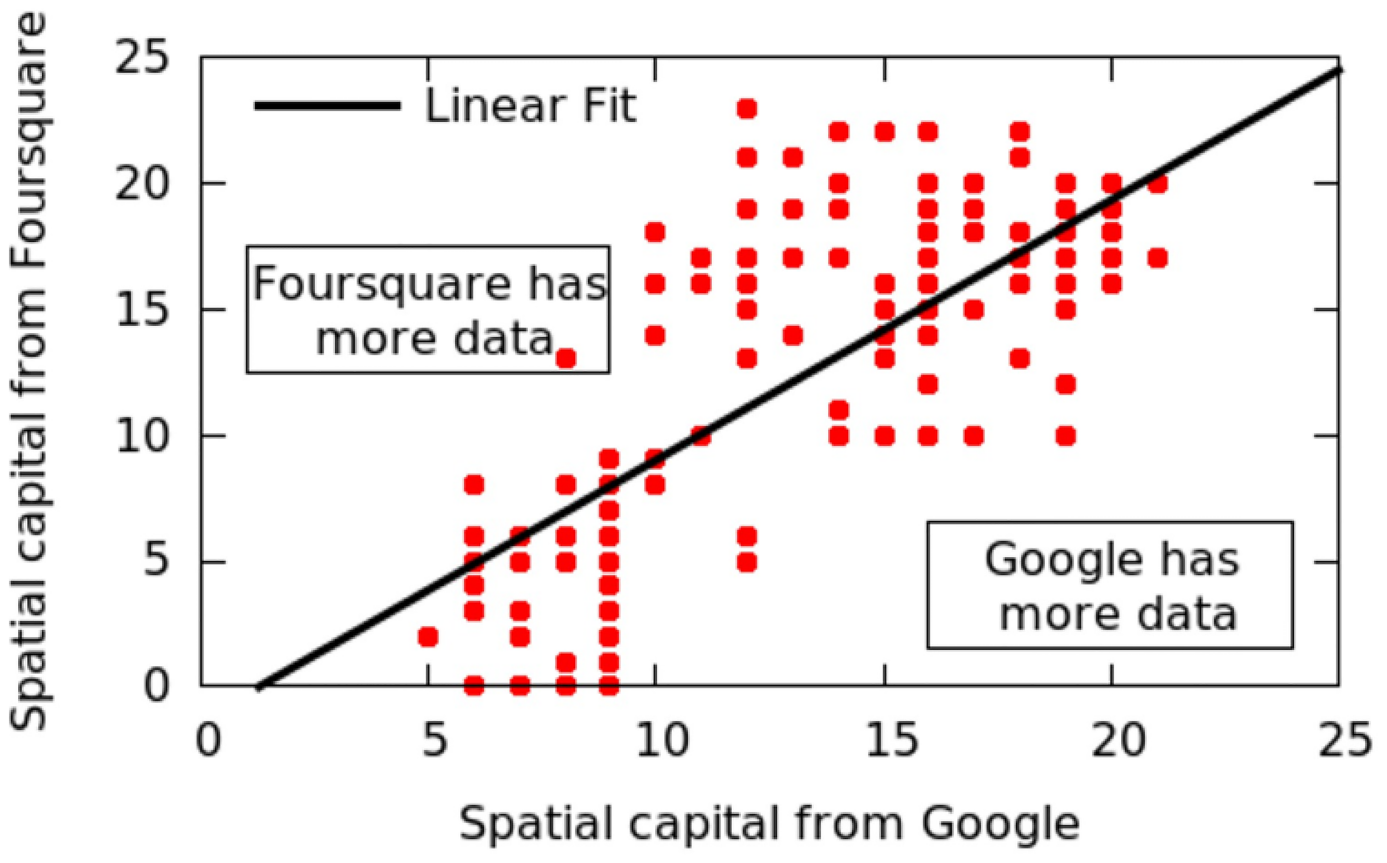
London spatial capital values computed upon Foursquare data (*y*-axis) *vs*. those computed upon Google data (*x*-axis). The Pearson Correlation is 0.77.

We have opted for Google Places data as it enjoys higher penetration rates than other online datasets. Figs 1 and 2 compares the number of amenities per category on Google with those on Foursquare and those on Open Street Map in the two cities of London and Delhi. The number of amenities per category in Google is far higher than Open Street Map’s and Foursquare’s, all the more so in Delhi. Despite these differences, our spatial capital metric does not change very much. Fig 3 shows the values of spatial capital computed on Foursquare data (*y*-axis) versus those computed on Google data (*x*-axis) in London: the Pearson Correlation is as high as 0.77, suggesting that the social capital metric is fairly robust against differences across spatial datasets.

## 3 Results

### 3.1 Urban interventions

With the place data at hand, we test whether our metric of spatial capital could inform urban interventions. More specifically, we study which amenity categories currently missing in an area would make a considerable difference, if they were to be added.

*Step 1: Identifying haves and have-nots*. Each area is characterized by its *C*_*a*_, which reflects the extent to which *a* offers many and diverse amenity categories at walking distance. Because of spatial auto-correlation, nearby areas tend to share similar spatial capital. Therefore, before any analysis, to control for spatial auto-correlation, we cluster areas that are nearby and similar by running the *K* means clustering for different values of *K*. Based on cluster silhouettes, the best values of *K* are between 4 and 8.

**Fig 4:**
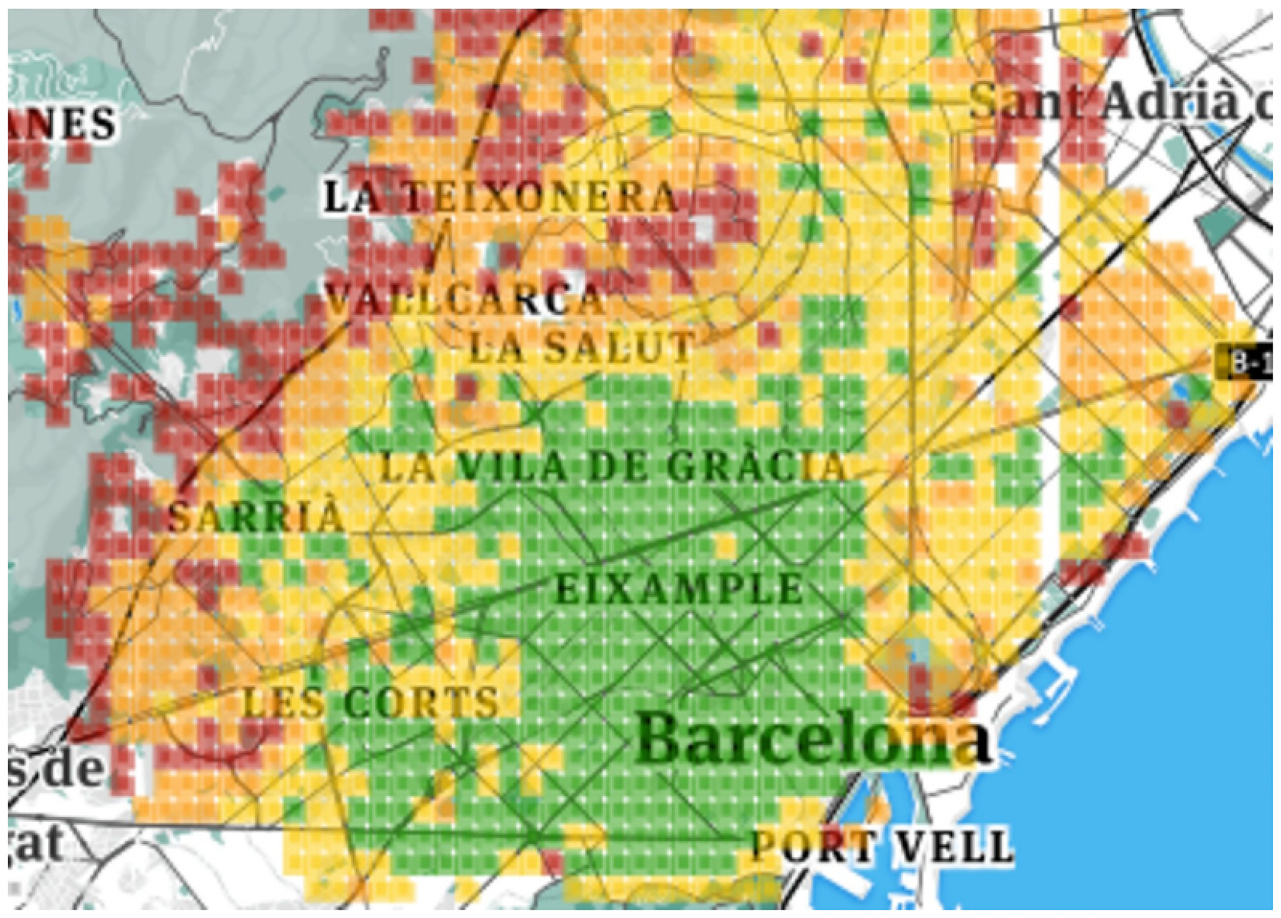
Barcelona four clusters.

**Fig 5:**
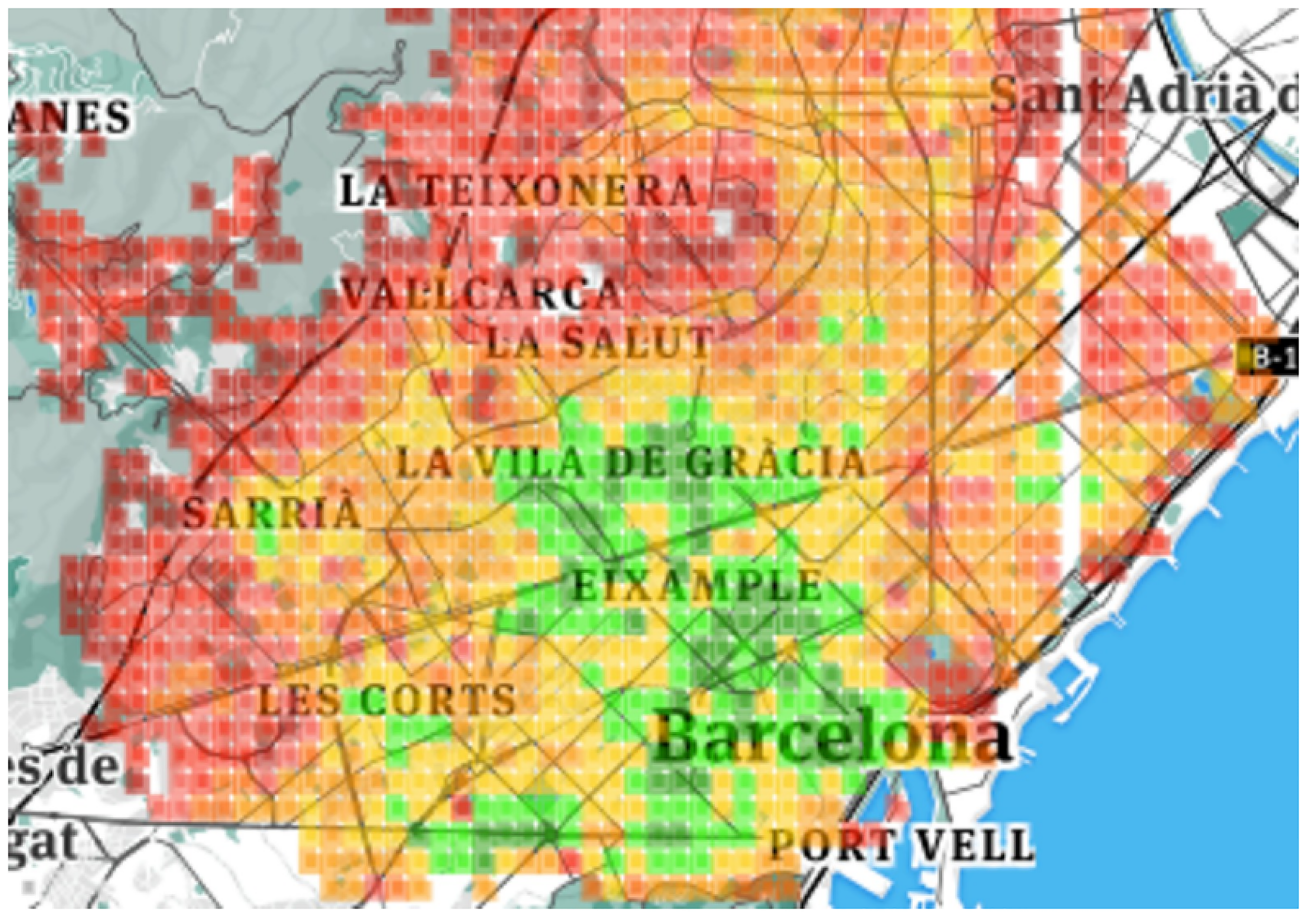
Barcelona eight clusters.

**Fig 6:**
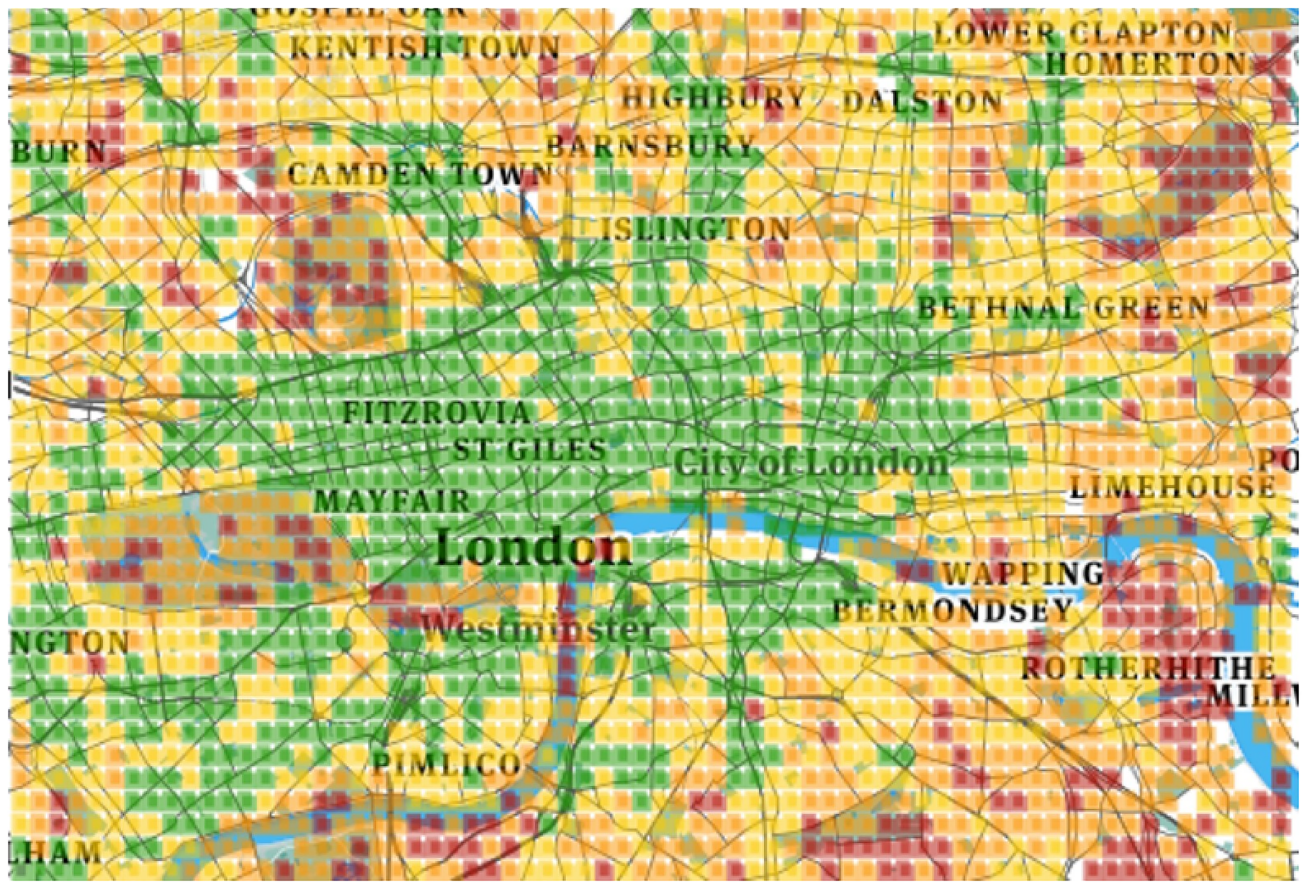
London four clusters.

**Fig 7:**
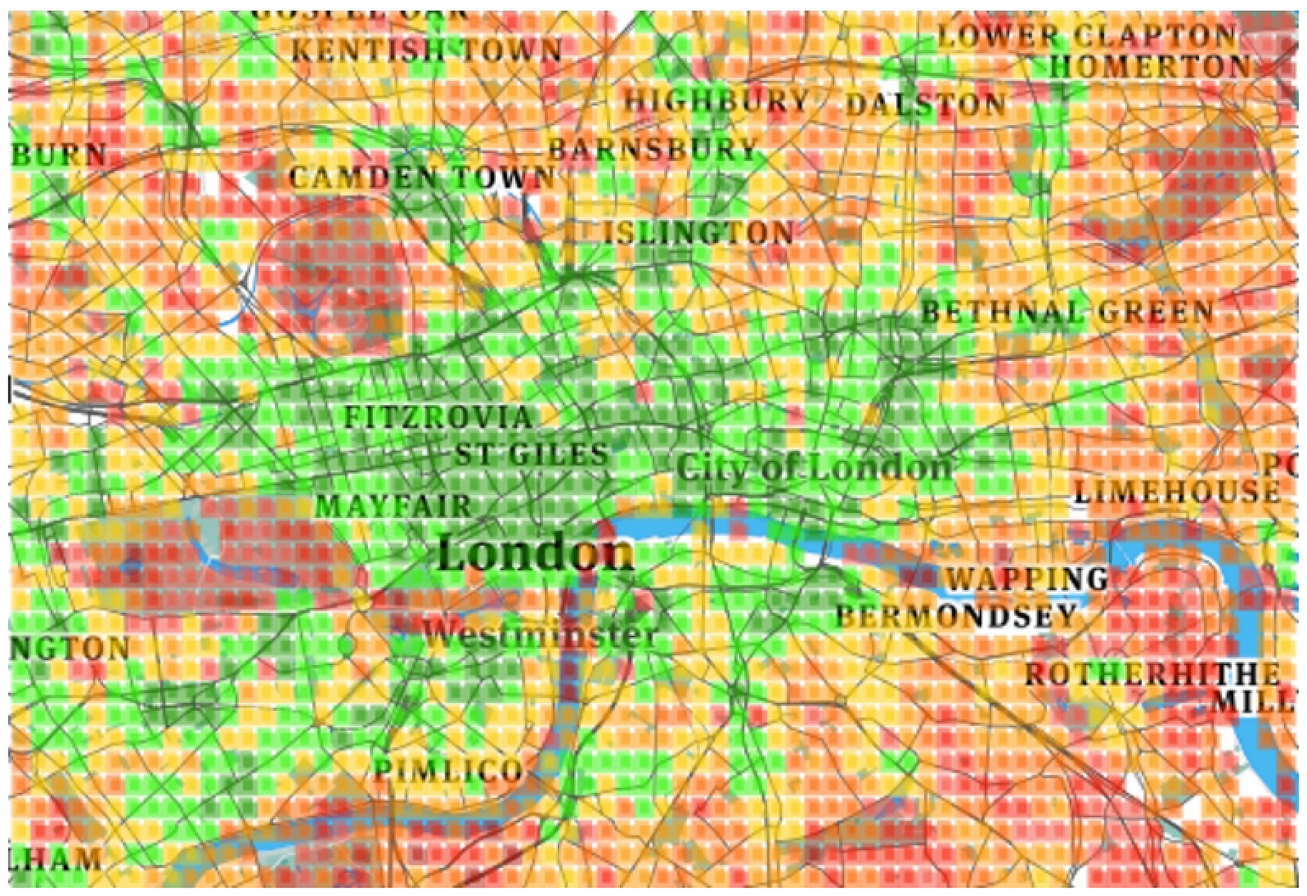
London eight clusters.

By visually inspecting Figs 4, 5, 6 and 7, one can see that for both *K* = 4 and *K* = 8, the resulting clusters are informative. To see why, consider that amenities are easily accessible in green areas, moderately accessible in yellow ones, and poorly accessible in red ones. As one expects, in Barcelona, good conditions are enjoyed by the center of the city, while poor conditions are confined to city boundaries. The same goes for London: good conditions are more prevalent in the center. As opposed to Barcelona, however, London shows worse conditions (denoted by orange and red colors) here and there. This difference comes from the way urban activity is organized. As we will show in the next section, London tends to be more poly-centric than Barcelona.

**Fig 8:**
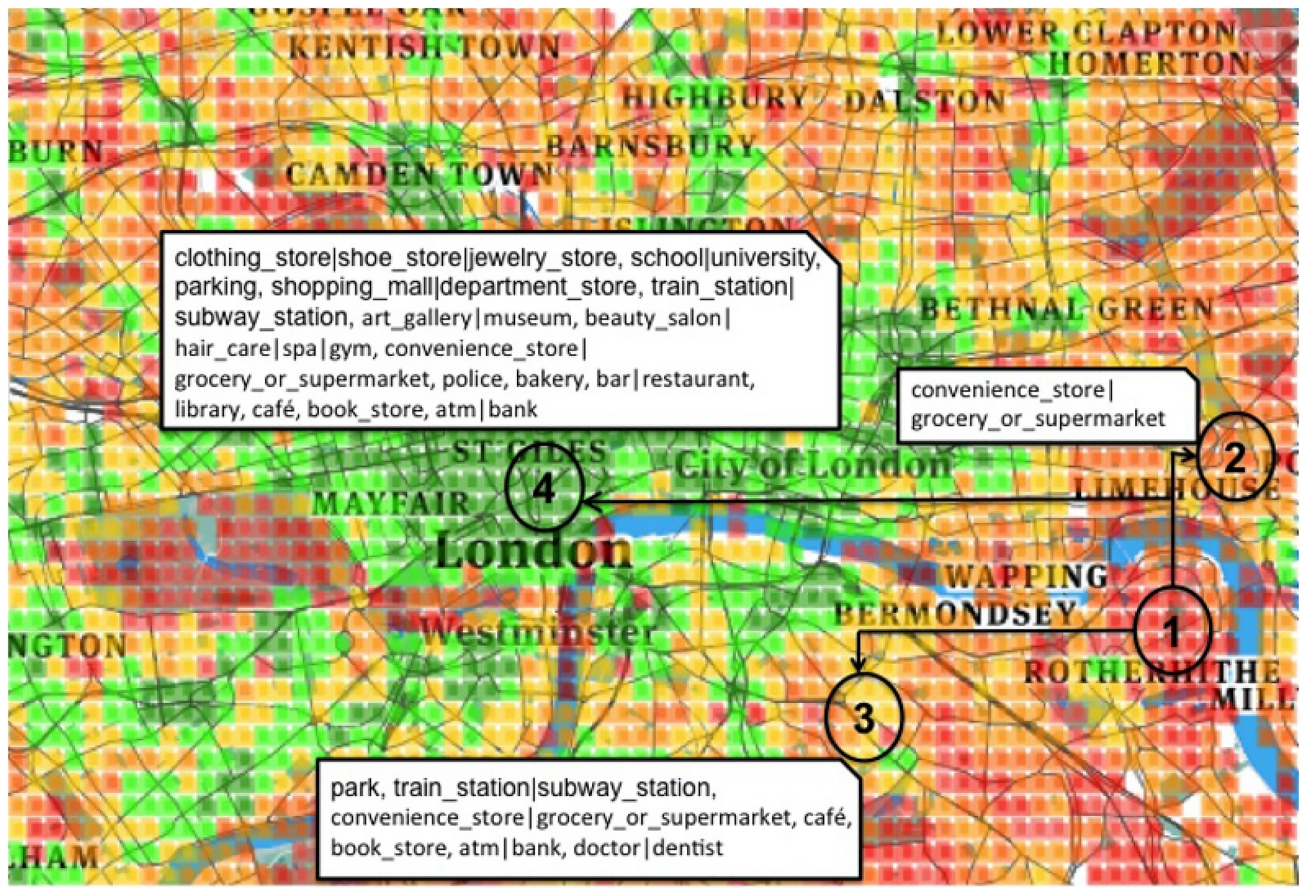
The white boxes next to areas (2), (3), (4) list the categories that are necessary for the red area (1) to become as accessible as the orange area (2), the yellow area (3), and the green area (4).

*Step 2: Compare haves against have-nots*. After clustering similar areas together, it is then possible to see which areas might benefit from the addition of new amenities. For example, consider the four London areas numbered in Fig 8. Area 1 is red, lies at the outskirt of Rotherhithe (East London), and offers access to amenities in a very limited number of categories. To become similar to the orange area (area 2), it would need to just add a grocery shop. To then become similar to the yellow area (area 3 in South London), it would need to add a grocery shop, a subway station, and a few amenities for daily errands. Finally, to become similar to the green area (area 4 in Central London), it would need considerable investments. The first two steps may represent interventions that not only increase spatial capital but also could be done cheaply.

### 3.2 Polycentricity for planning

Urban planners have long examined the question of whether a city is mono- or poly-centric. Poly-centric cities are those that have dispersed activity zones, while mono-centric cities are those that grow from a core municipal area [17, 18]. Knowing a city’s centers allows planners, for example, to inform evidence-based interventions that reduce travel times. That is why digital data has already been used to study urban poly-centricity depending on Foursquare activity [13], and on commuting activity in London [19].

#### 3.2.1 Visualizing poly-centricity

**Fig 9:**
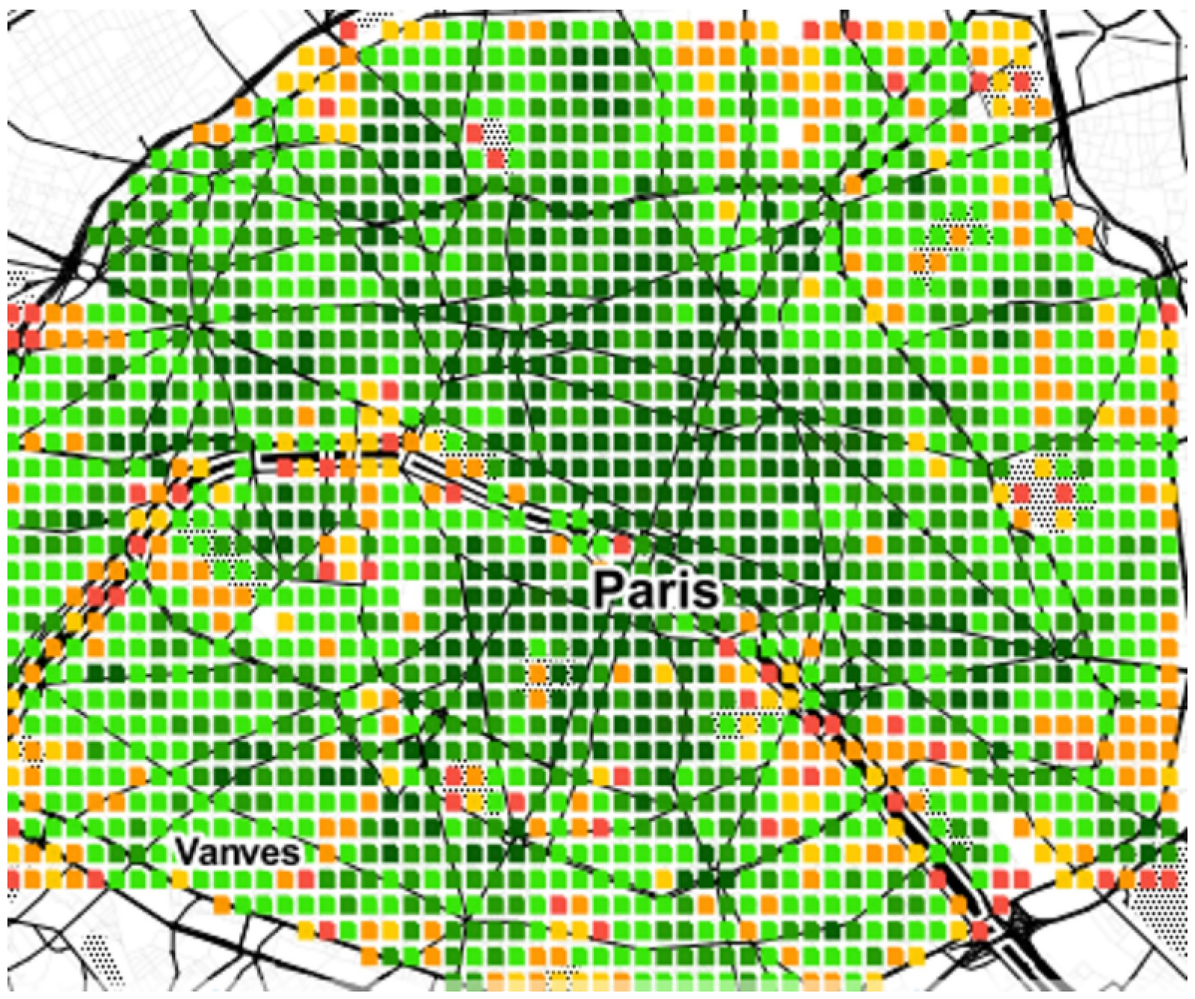
Mono-centric Paris.

**Fig 10:**
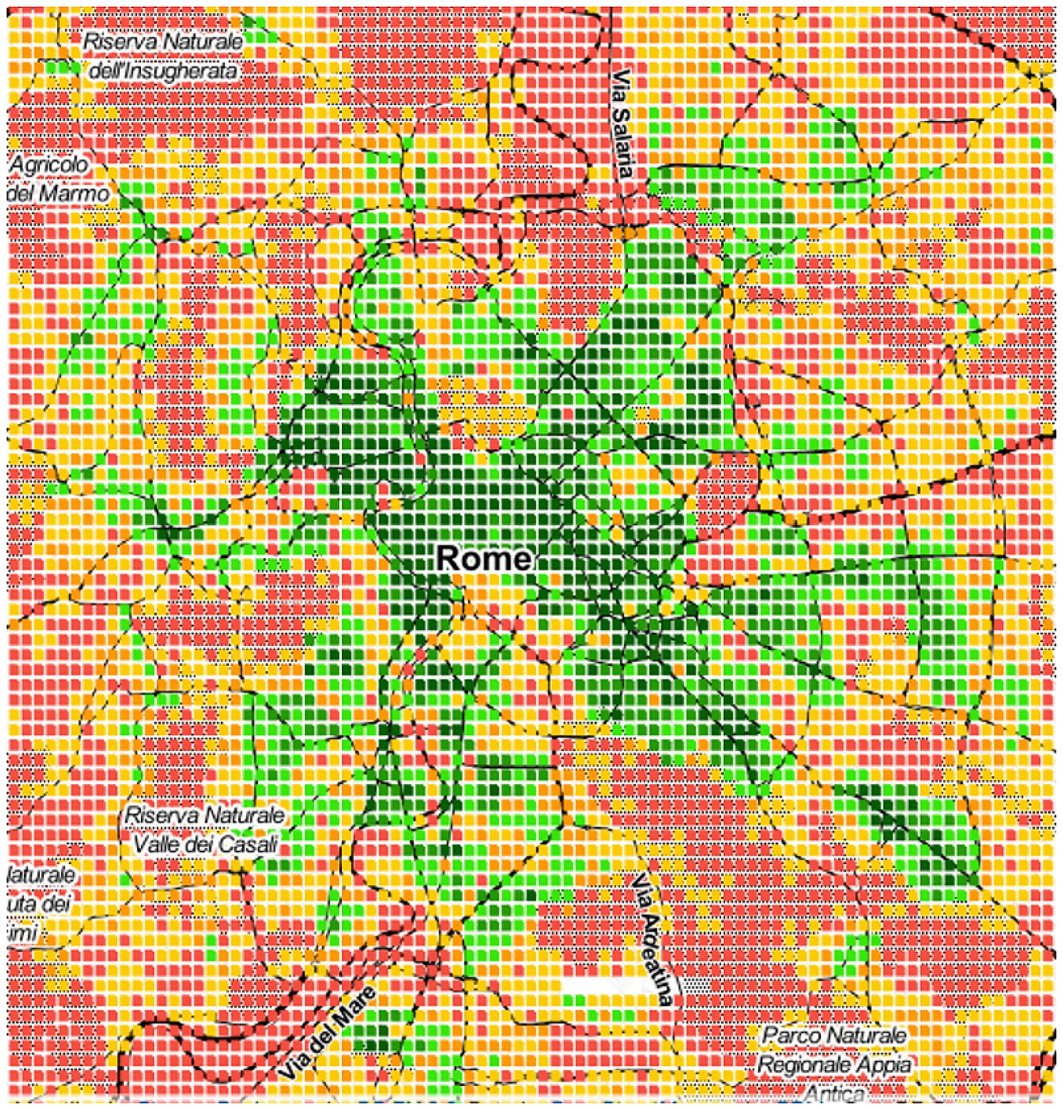
Mono-centric Rome.

**Fig 11:**
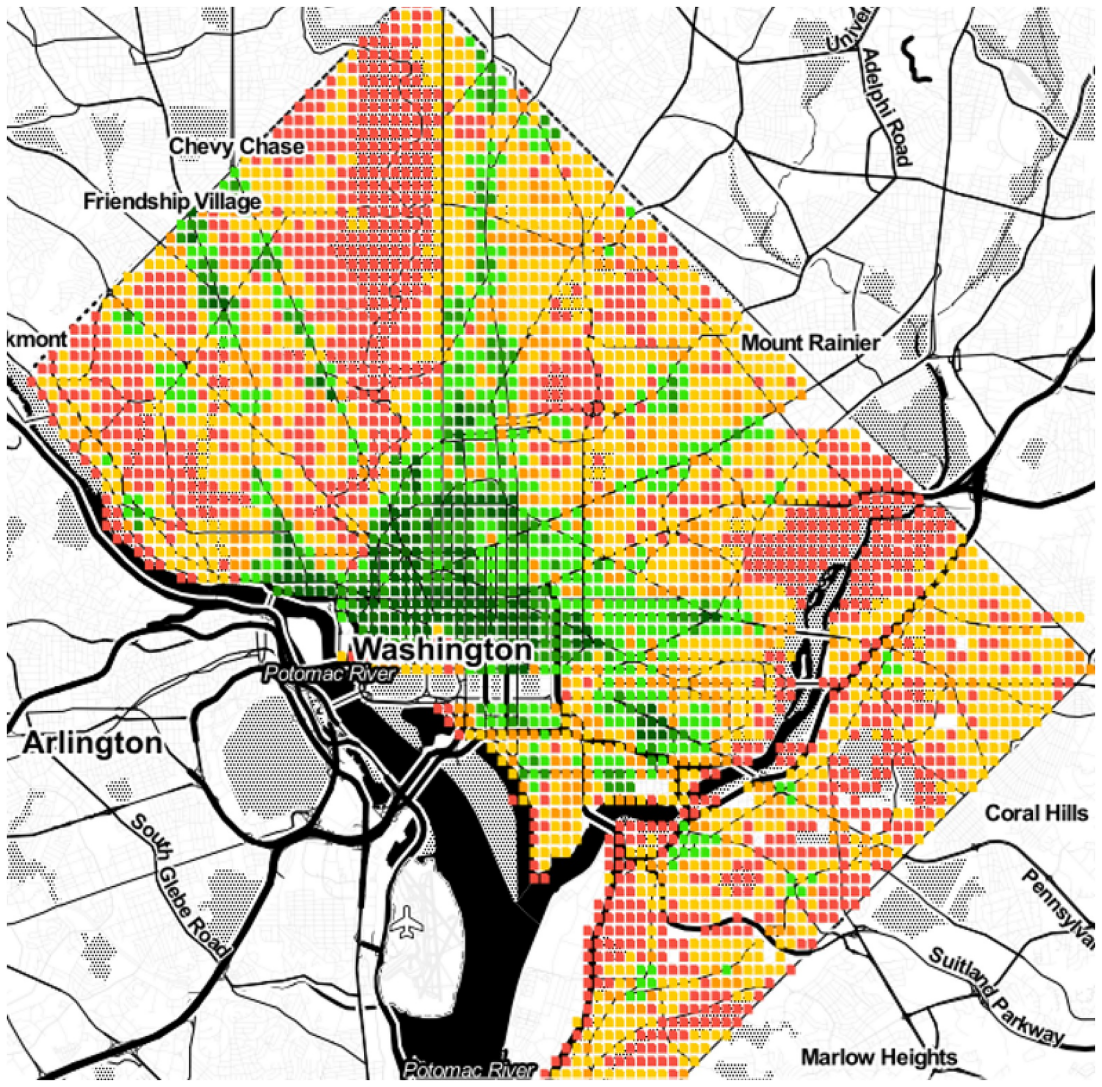
Mono-centric Washington.

**Fig 12:**
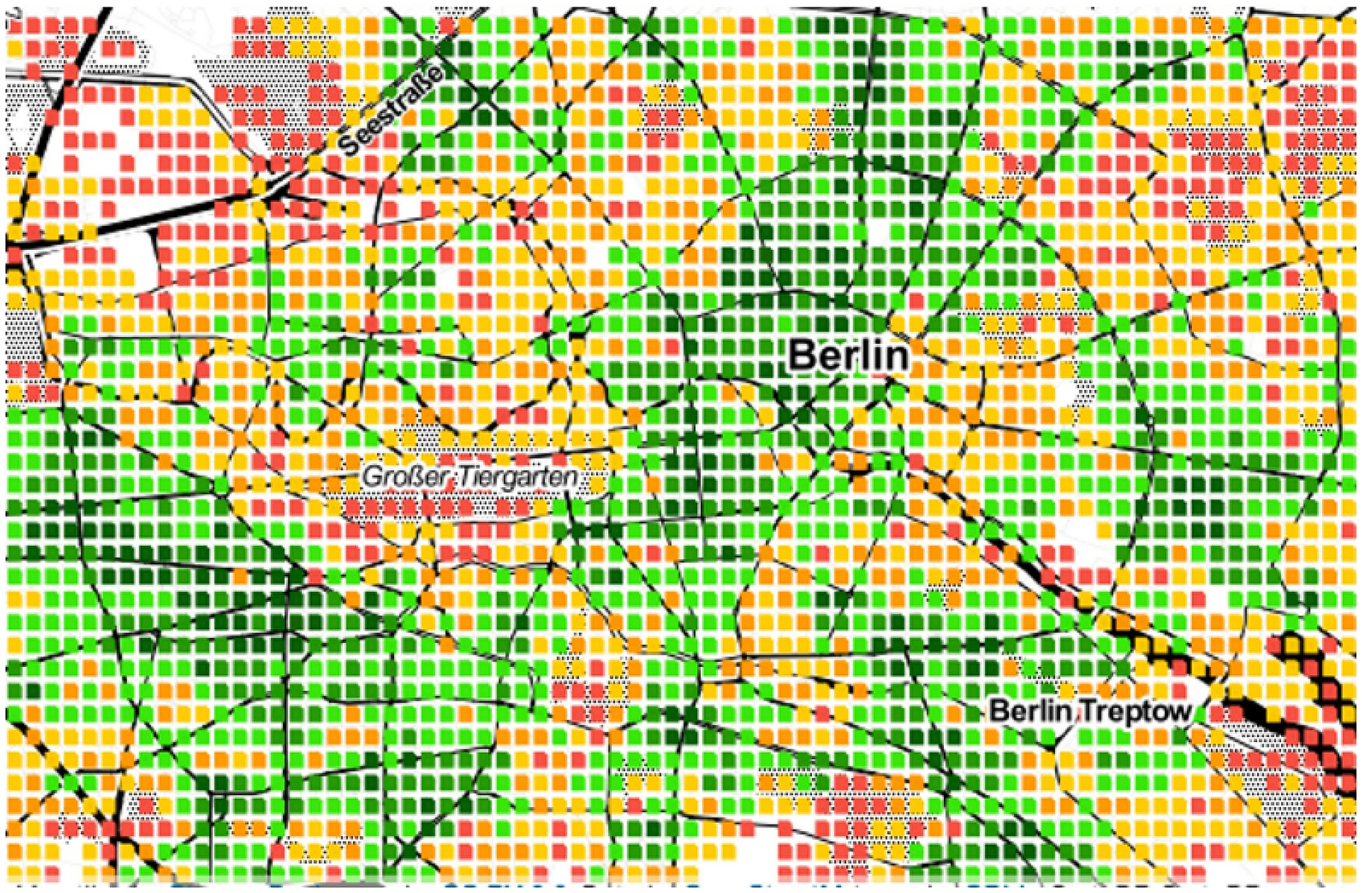
Poly-centric Berlin.

**Fig 13:**
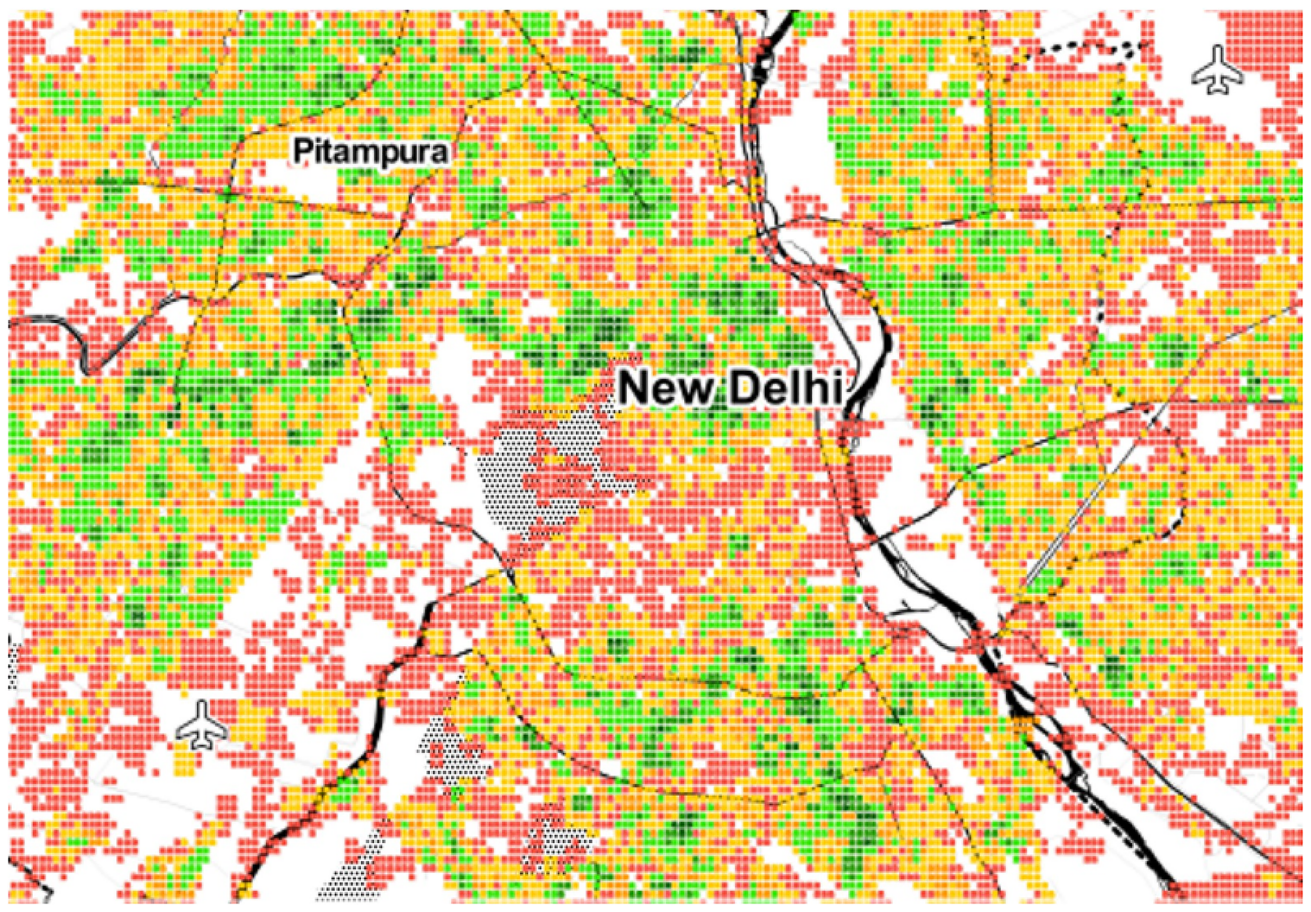
Poly-centric Delhi.

**Fig 14:**
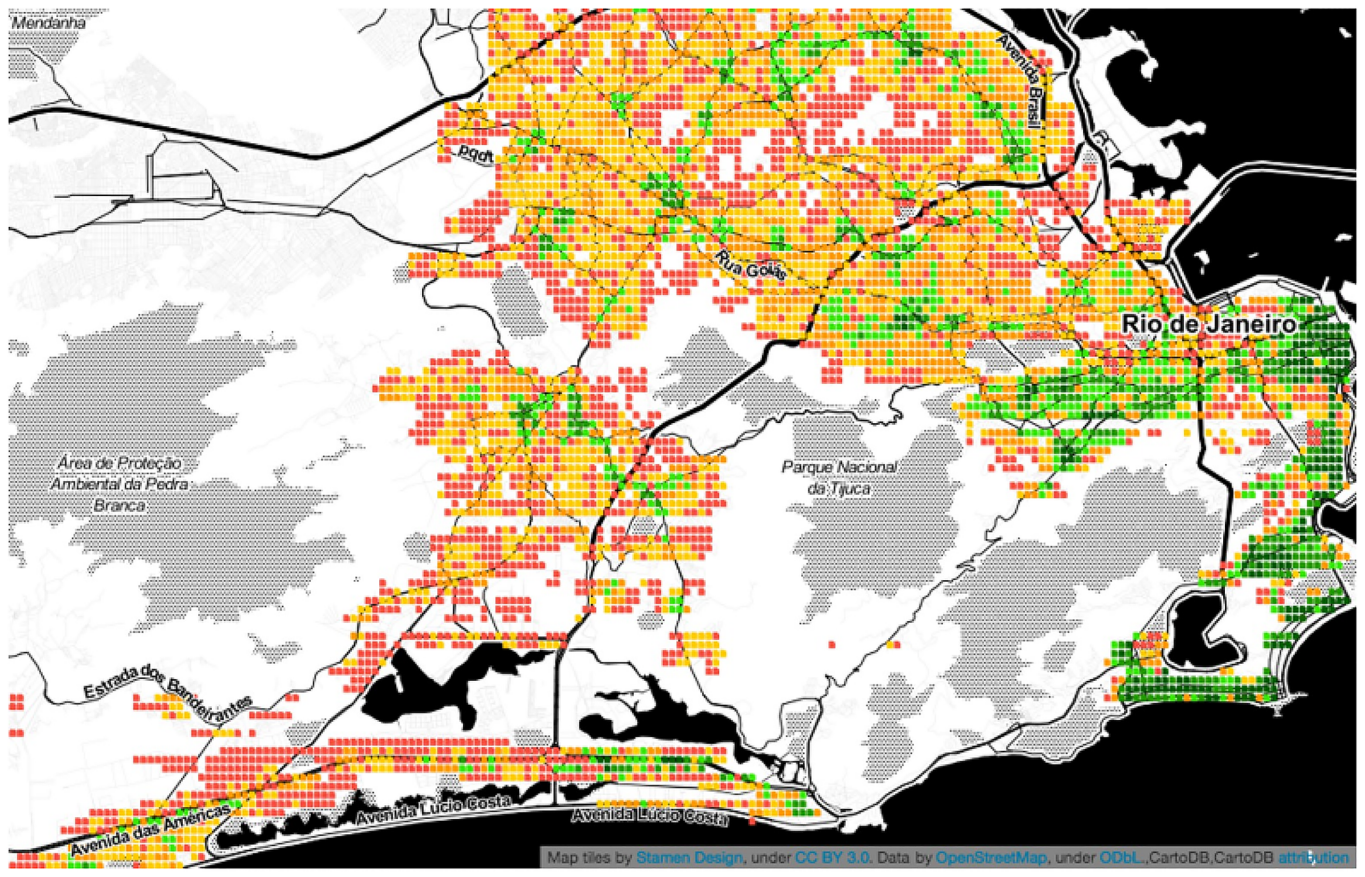
Poly-centric Rio.

As we have seen, area *a*’s spatial capital *C*_*a*_ reflects how many categories *a* has at walking distance. We now map those values to ascertain whether one could visually determine the extent to which a city is mono- or poly-centric. Given the limited space, the maps of only six cities are shown in Figs 9 to 14. The six cities have been chosen because they are quite diverse from each other. Darker shades of green represent higher values of *C*_*a*_, while progressively worse values are in yellow, orange and red.

From the map, we see that Paris, Rome and Washington are mono-centric since accessible areas tend to cluster together. By contrast, Berlin, Delhi and Rio De Janeiro are poly-centric since accessible areas tend to be here and there.

Those observations are in agreement with those produced by other researchers who used data and methodologies different than ours, speaking to the external validity of our metric and methodology. By studying three European cities, Marull *et al*. found Paris and Rome to be mono-centric, while they found Berlin to have a reticular or net-like poly-centric structure [20]. Arribas-Bel *et al*. focused on Washington and found it to be mono-centric [21], as we just did.

No prior study has been conducted for cities in the developing world like Delhi and Rio De Janeiro. We were able to study them here and, by visually inspecting the two maps, one can clearly see that activity is bounded by geographical barriers: by the Yamuna river in Delhi, and by the national park of Tijuca in Rio. These natural barriers end up splitting urban areas into disjoint poly-centric structures.

#### 3.2.2 Quantifying poly-centricity

To go beyond the mapping of poly-centricity, we move onto quantifying it. If areas of high spatial capital are all close together in a city, then the city tends to be mono-centric. Alternatively, if those areas are dispersed, then the city tends to be poly-centric. To quantify that, we compute the metric *μ*, which can be thought as spatial dispersion (a spatial “standard deviation”):

$$\begin{array}{c} \mu =\sqrt{\sum_{a=1}^{N}\frac{C_{a}\cdot d_{a}^{2}}{C}} \end{array}$$ (2)

To see how the numerator works, imagine to order the city’s areas by decreasing values of spatial capital *C*_*a*_. By considering the top*N* most accessible areas and then determining their mean centroid, *μ*’s numerator weighs area *a*’s spatial capital (*C*_*a*_) by its distance to the city’s centroid (*d*_*a*_). The intuitive explanation behind the denominator is that, if the top *N* areas are close together, then their mean centroid lying in an intermediate position will cause all *d*_*a*_ values to be small and, as such, *μ* will have a small value. On the other hand, if the top *N* centers are spatially dispersed far apart, their mean centroid in an intermediate position will give large values for *d*_*a*_, causing *μ* to be large. The denominator (*C* = ∑_*a*_ *C*_*a*_), on the other hand, normalizes the numerator by the sum of spatial capital values for the top*N* areas. This normalization makes *μ* comparable across cities as it accounts for city-specific values of *C*_*a*_: cities in the developed world might have much higher values of *C*_*a*_ than those in the developing world.

**Fig 15:**
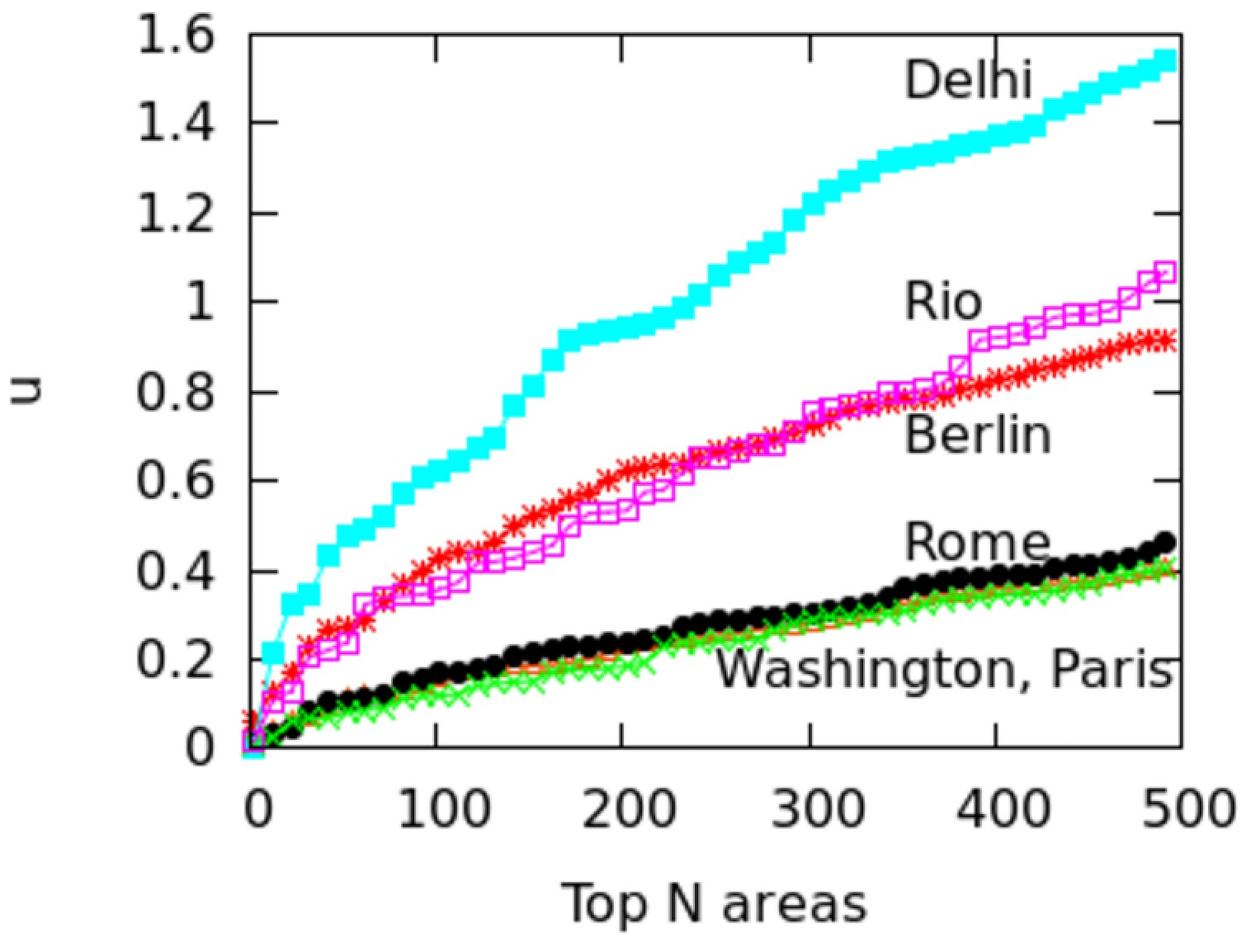
*μ* values for top *N* = 2‥500 areas in three mono-centric and three poly-centric cities.

By considering an increasing number *N* of areas and ordering them by decreasing *C*_*a*_, it is possible to plot the center dispersion graph (Fig 15). This shows how *μ* on the *y*-axis changes when an increasing number of top*N* areas is considered. A steep rise means that the top*N* areas are spread apart (typical of poly-centric cities), while a gradual rise means that the top*N* are geographically clustered (typical of mono-centric cities). For *N* from 2 to 500, we see results similar to those visually observed on the maps of Figs 9 to 14: Paris, Rome and Washington are less poly-centric than Berlin, Delhi and Rio. *μ*’s steepness can be further quantified in terms of average curve slope *m*:

$$\begin{array}{c} m=\frac{\sum_{k=1}^{N-1}\mu_{k+1}-\mu_{k}}{N-1} \end{array}$$ (3)

Then, the list of cities ranked by *m* is effectively an ordered list by degrees of poly-centricity (Table 3): Delhi is far more poly-centric than Washington.

**Table 3:** List of cities inversely ranked by degree *m* of poly-centricity. *m* is the average slope of *μ* and, for readability, it is rescaled by a multiplication factor of 10^3^.

City	*m* ⋅ 10^3^
Washington	0.792
Paris	0.808
Rome	0.929
Berlin	1.725
Rio	2.234
Delhi	3.131

**Fig 16:**
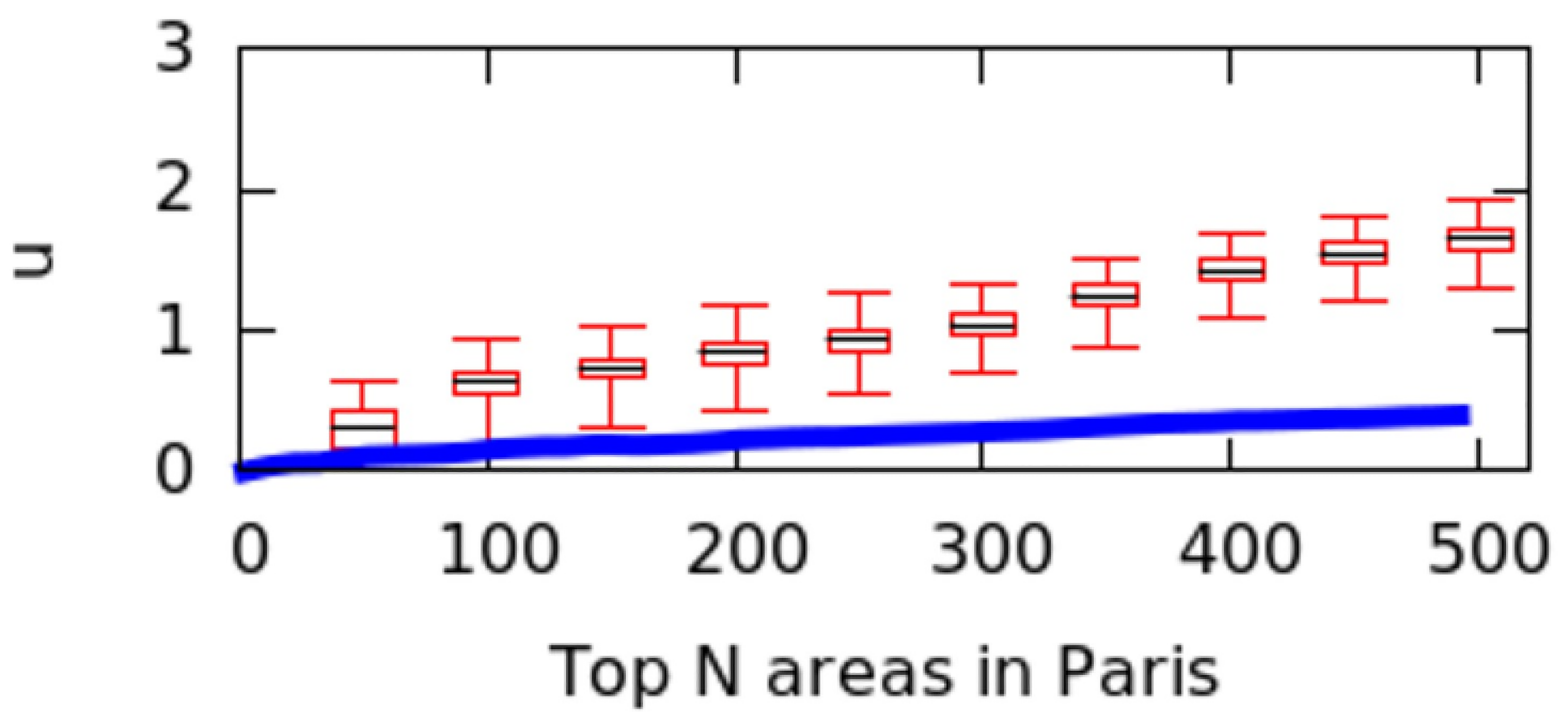
Null model in Paris.

**Fig 17:**
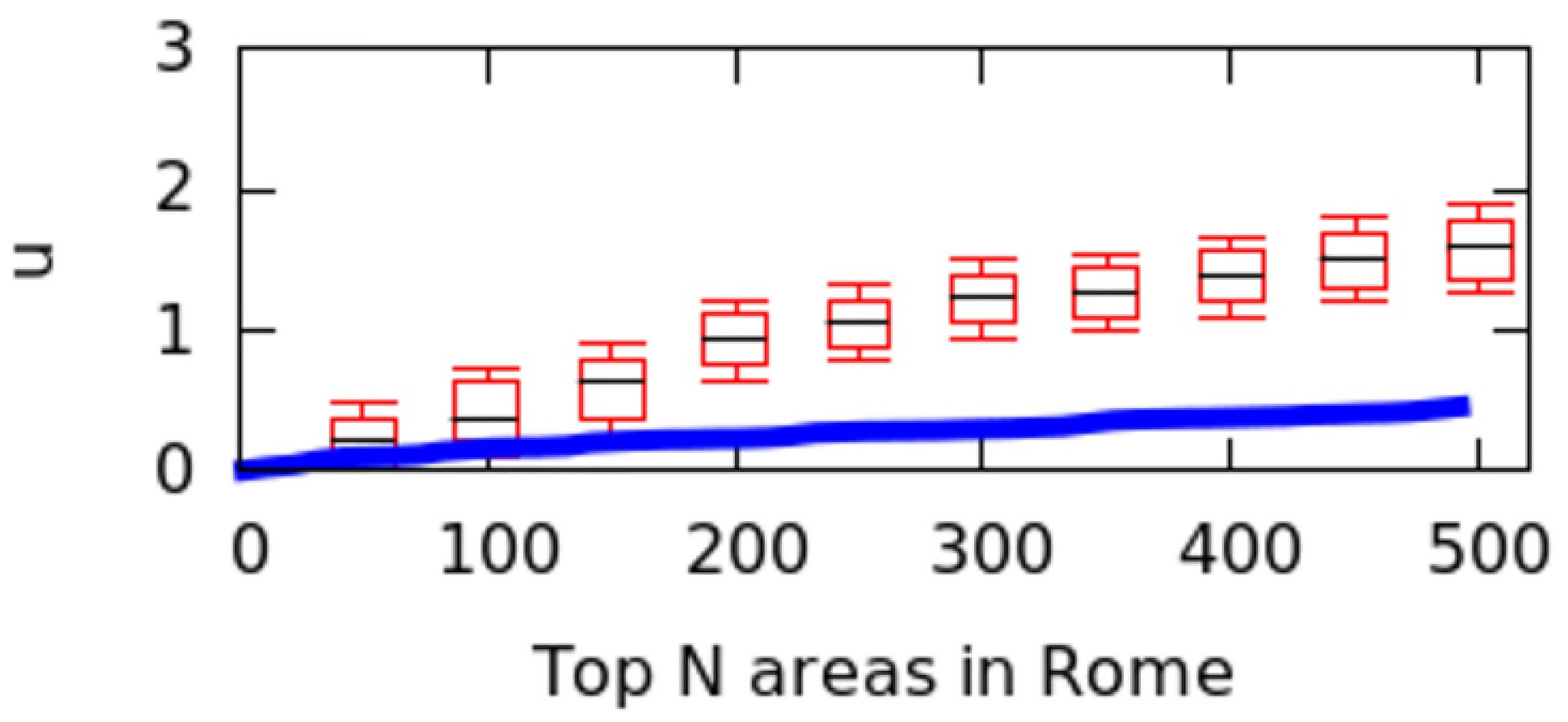
Null model in Rome.

**Fig 18:**
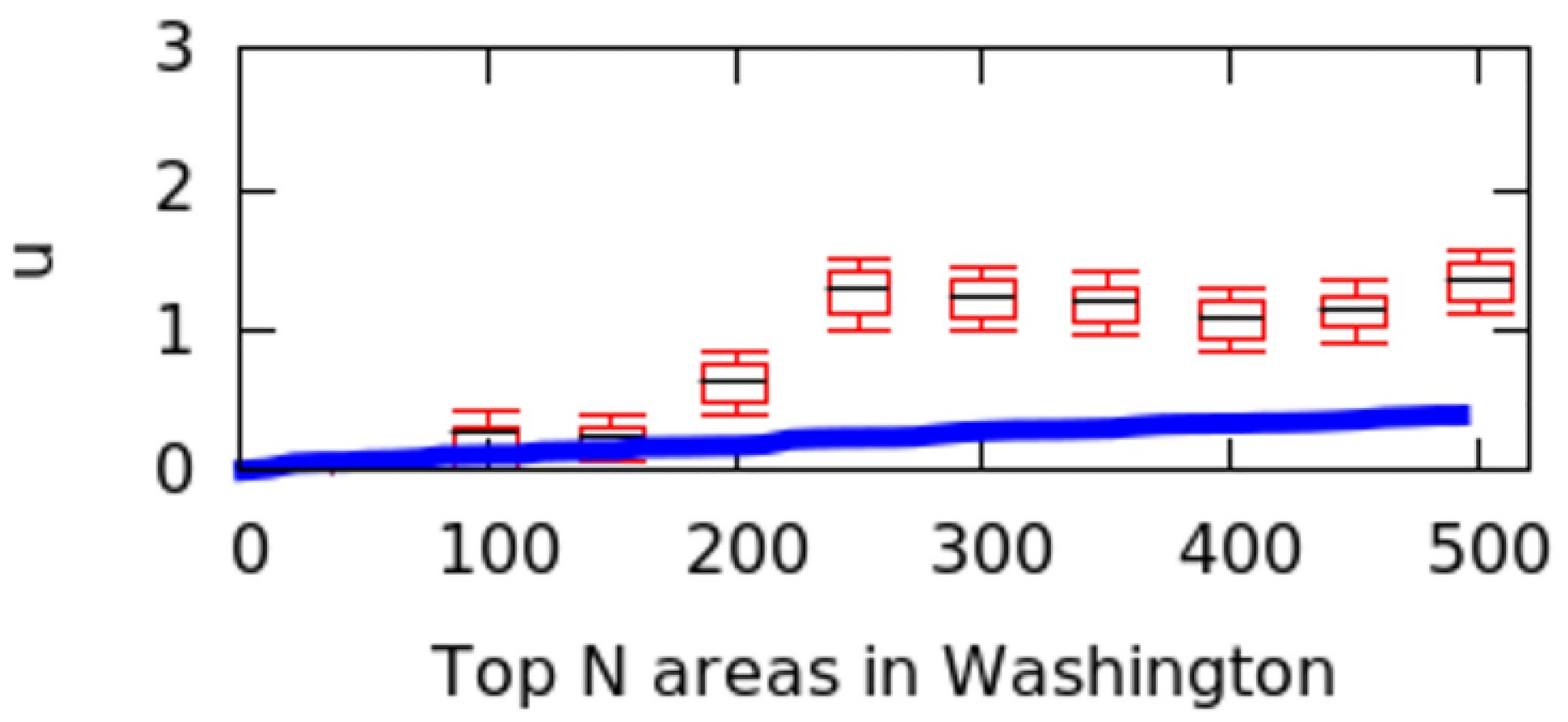
Null model in Washington.

**Fig 19:**
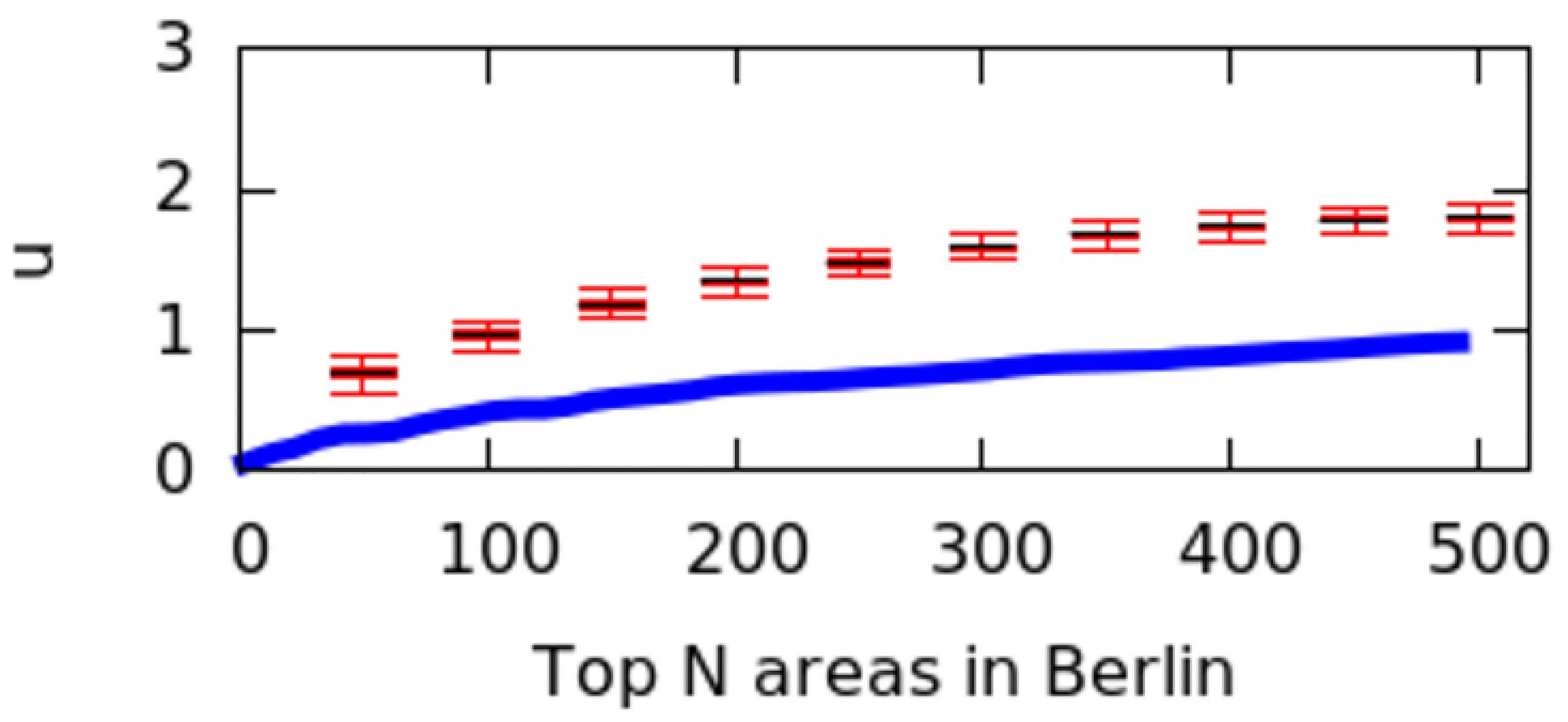
Null model in Berlin.

**Fig 20:**
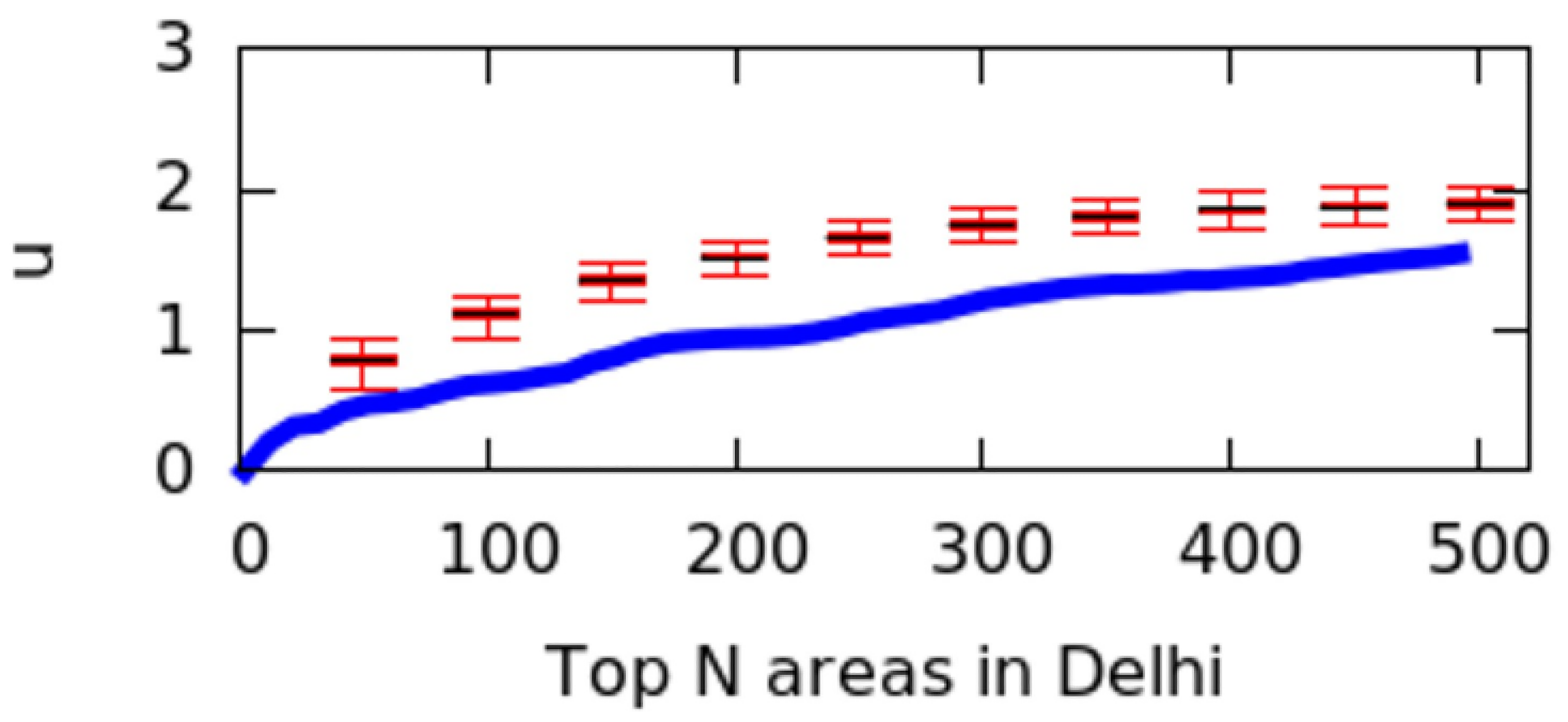
Null model in Delhi.

**Fig 21:**
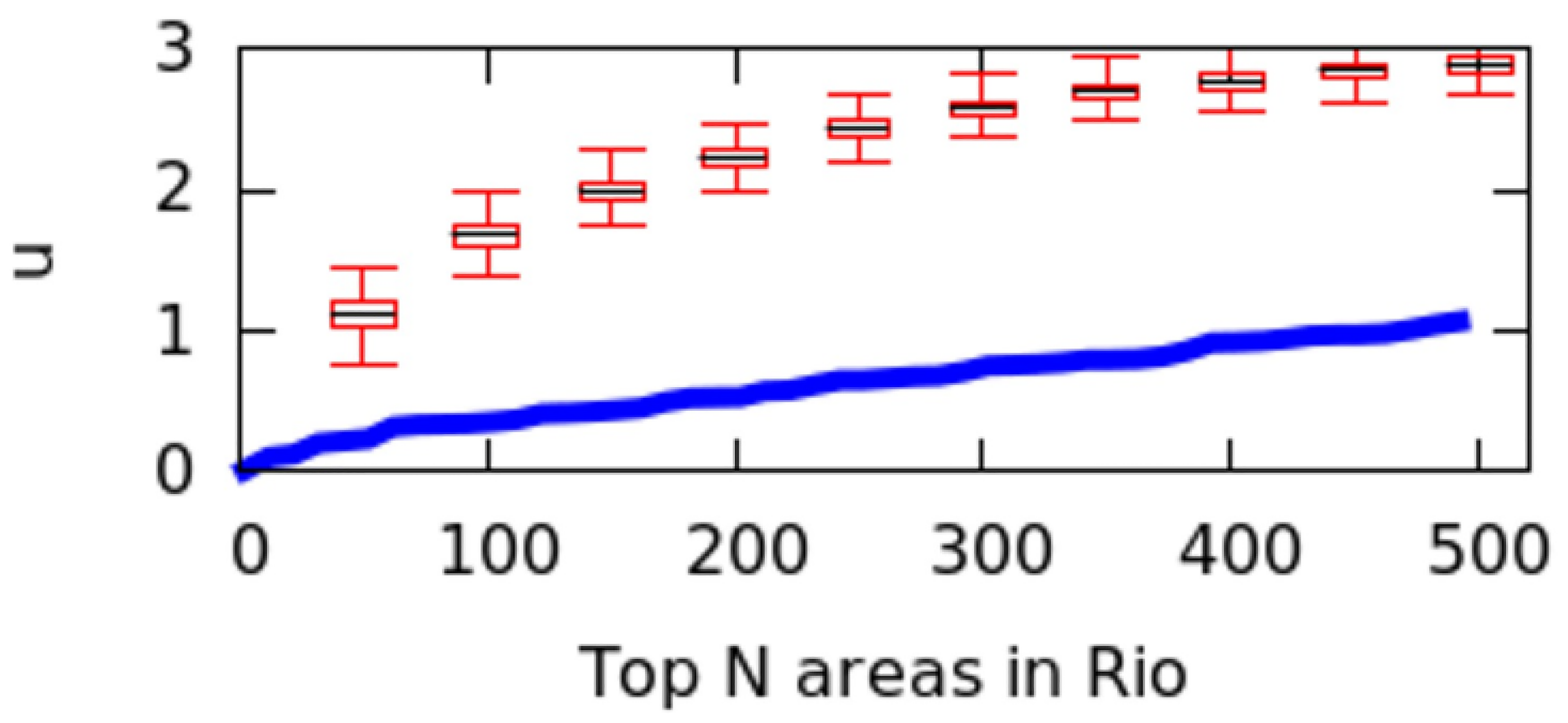
Null model in Rio.

However, city size might impact the calculation of *μ* values, and we have not controlled for it. That is because a big city might have geographical breaks (e.g., rivers, forests) that separate it into different regions, artificially causing poly-centricity. To establish whether the level of poly-centricity is guided only by geographical factors, the formulation of a null model is in order [22]. The null model should keep the city’s size and the position of the city’s geographical barriers constant and randomize the rest. In each city, this is achieved by randomly distributing the top*N* areas and recomputing *μ* each time. After doing so for more than 10,000 runs, we plot the quartile values of *μ* for the null model as box-plots for the six cities in Figs 16 to 21; for reference, the original values of *μ* are plotted in blue.

If the degree of poly-centricity would be caused by city size and geographical barriers alone, then the blue lines should overlap the box-plots. Instead, in reality, it is caused by other factors, including city planning. The gap between the blue line and the red one increases as, for example, more top-down planning (e.g., management of the historical legacy, zoning laws) took place. From Figs 16 to 21, one could infer that city-planning decisions have been made from the top down in Rio (widest gap), while Delhi’s growth appears to be more bottom up (smallest gap).

### 3.3 Comparing cities

**Fig 22:**
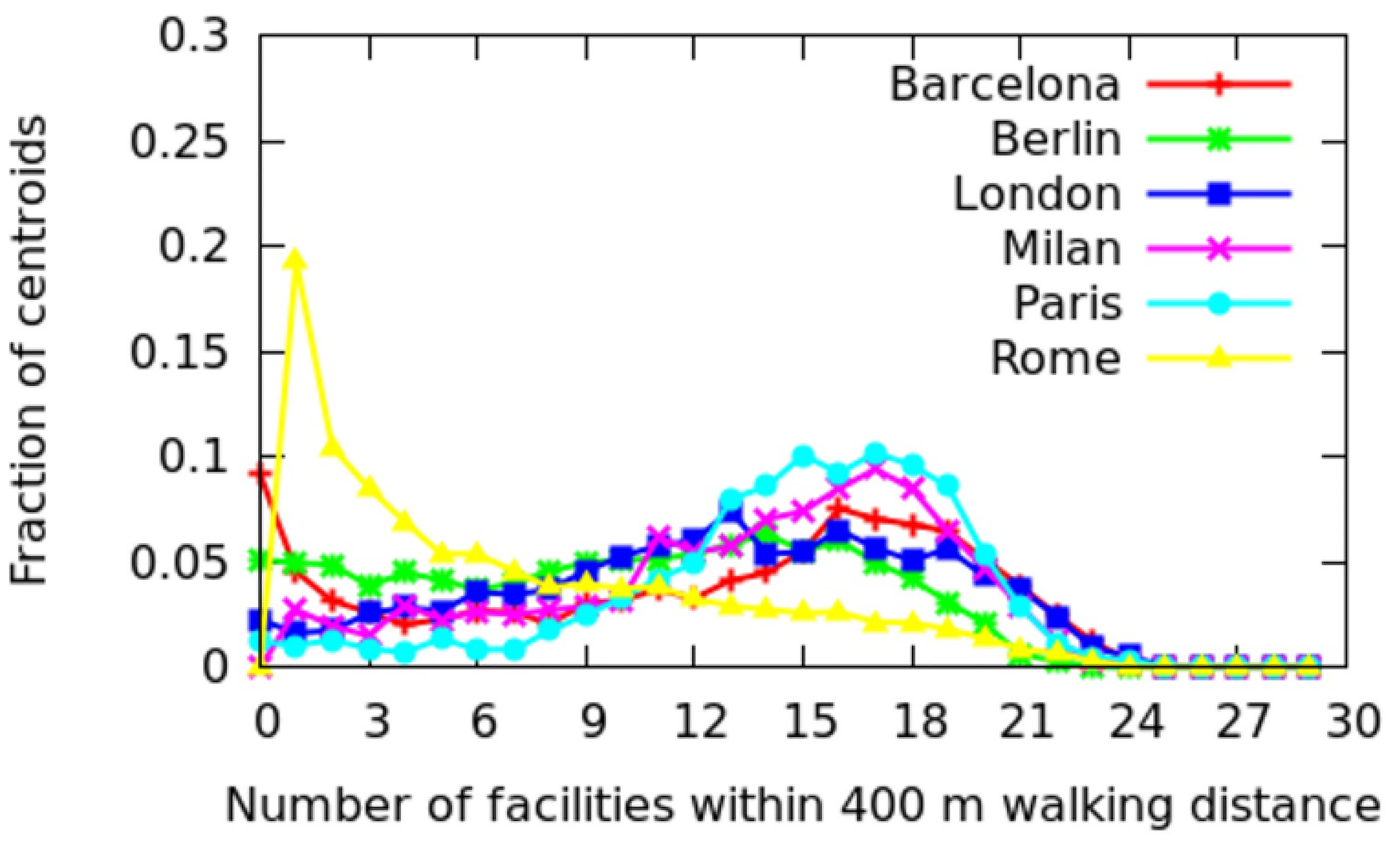
Fraction of areas with *x* categories at walking distance in Europe.

**Fig 23:**
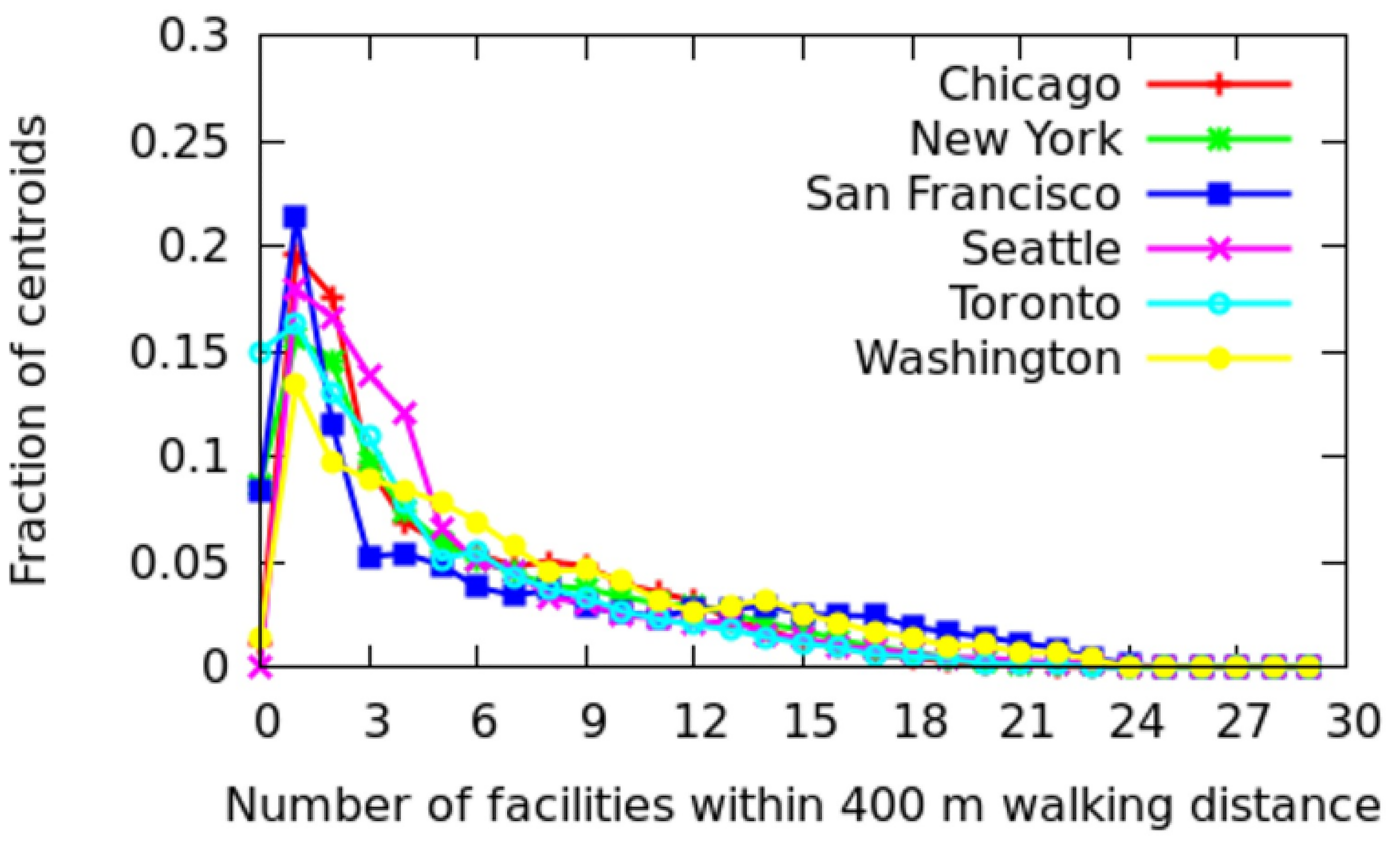
Fraction of areas with *x* categories at walking distance in America.

**Fig 24:**
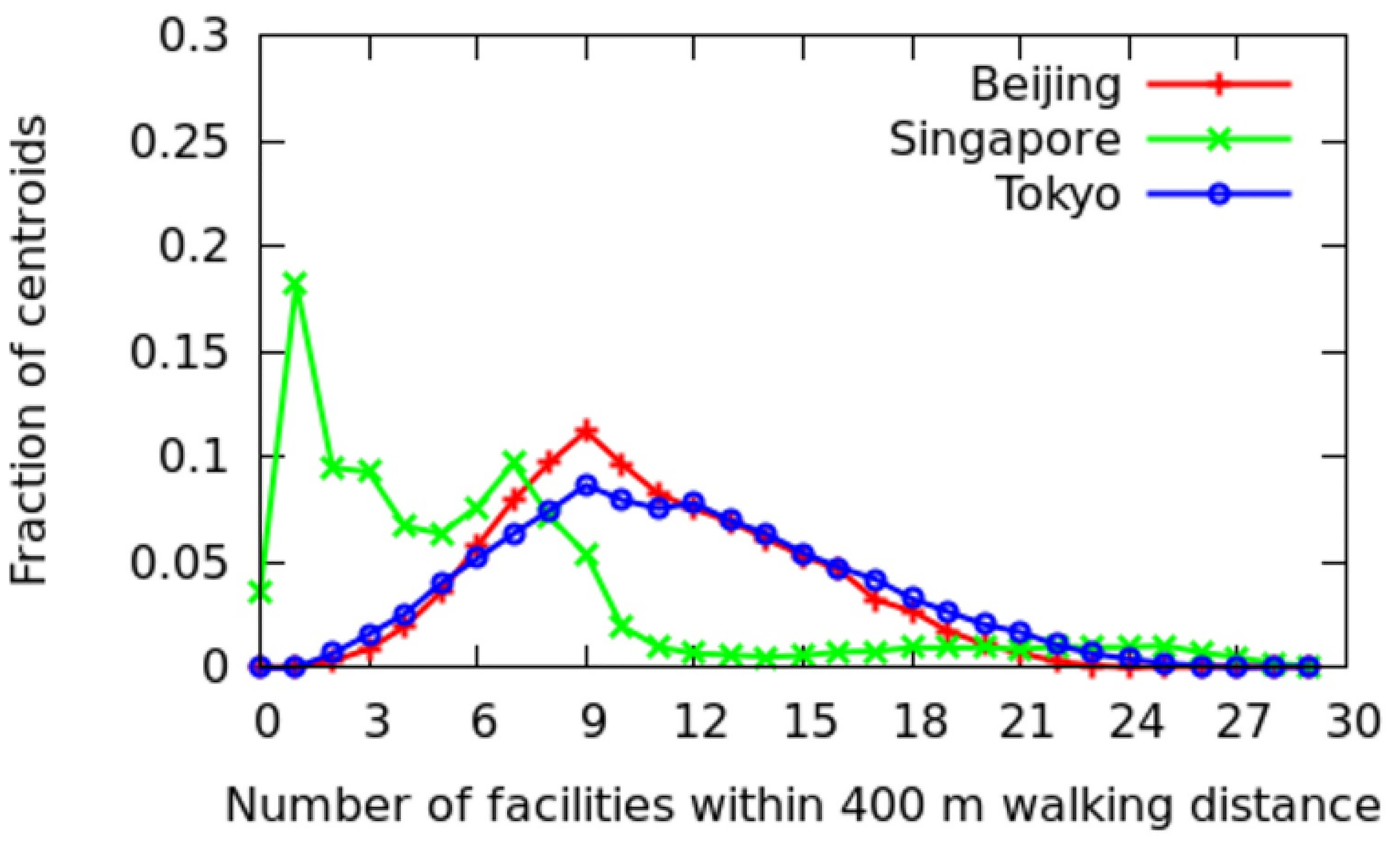
Fraction of areas with *x* categories at walking distance in Asia.

**Fig 25:**
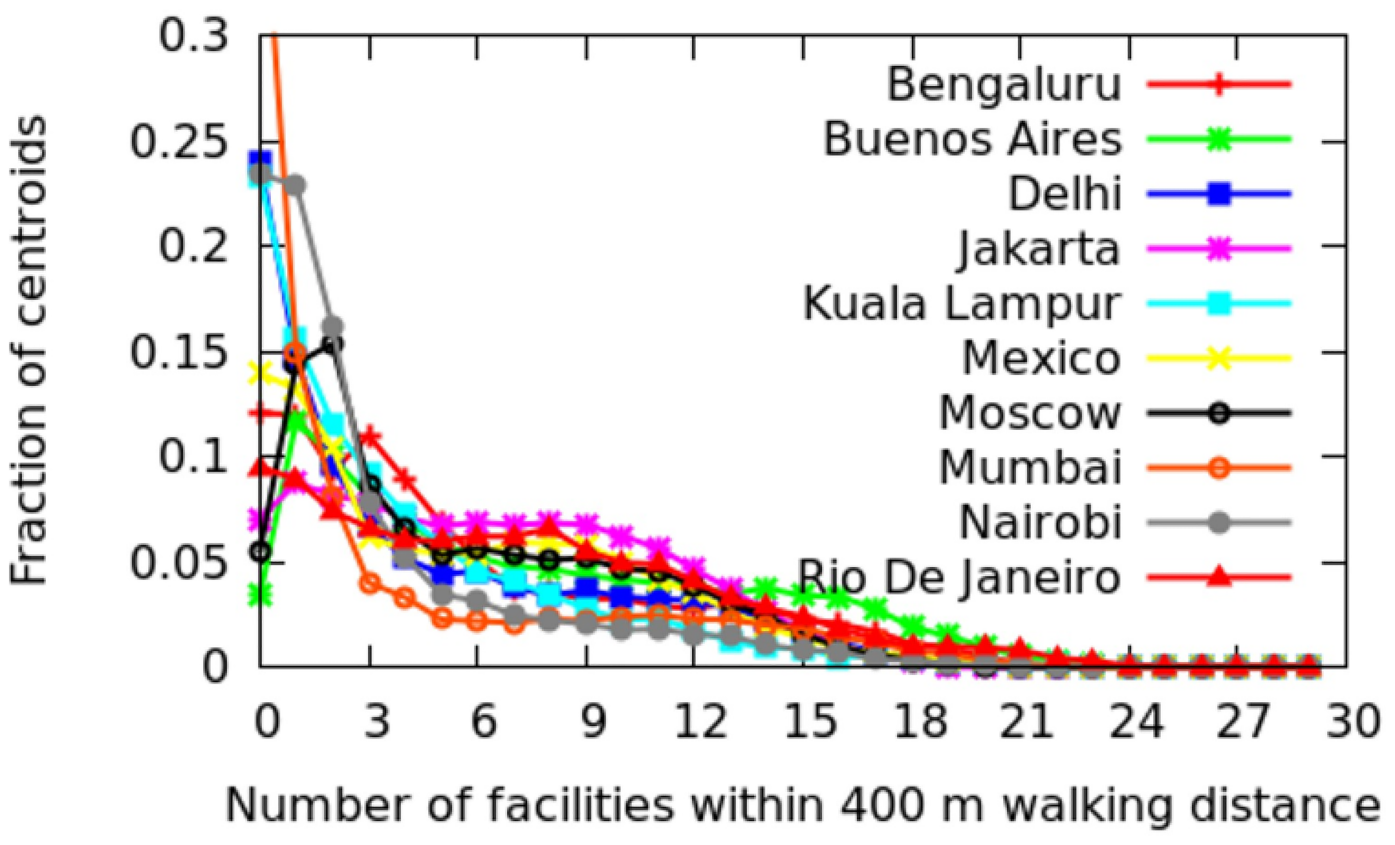
Fraction of areas with *x* categories at walking distance in developing countries.

In this section, we move from an intra-city analysis to inter-city analysis and compare all the twenty-five cities with each other. To ease illustration, the cities are grouped depending on whether they are in Europe, North America, Asia, and the developing world (Table 2). For each city, the fraction of areas that have *x* categories at walking distance (i.e., within 400 meters) are shown (Figs 22 to 25). More technically, we plot the probability density function (PDF) of *C*_*a*_. A peak on the right means that most of the areas offer good spatial capital, while a peak on the left means that most of the areas offer poor spatial capital. The *x*-axis goes from 0 to 30 as we have 30 categories in total.

*Europe*. In European cities (Fig 22), most areas enjoy good spatial capital (i.e., typically, 17 categories are at walking distance), and, for only a few areas, the spatial capital profile is bimodal: it is either very poor or very good. Therefore, all cities have peaks on the right, except Rome. As seen previously in Fig 10(b), Rome is strongly mono-centric: the center of the city (the ancient part) enjoys very good spatial capital, while the rest of the city does not.

*North America*. For North American cities (Fig 23), the peaks are on the left, suggesting that most areas offer a few categories at walking distance (typically 3 categories), and that results into the well-known car dependency [6, 7].

*Asia*. For Asia (Fig 24), Beijing and Tokyo are similar to European cities, although with slightly lower spatial capital (i.e., 9 categories are at walking distance). By contrast, Singapore shows two peaks (both on the left side): in most areas, either 2 or 7 categories are at walking distance, and there is little in between. That is because of Singapore’s zoning laws [8]: they end up to separate the residential, commercial, industrial, and port areas of the city.

*Developing World*. Cities in the developing world (Fig 25) show slightly worse spatial capital than cities in North America (i.e., typically only 2 categories are at walking distance). Buenos Aires is an exception: a considerable number of areas have as may as 17 distinct categories at walking distance. In previous studies, it has been found that Buenos Aires is indeed walkable [9], so much so that it was granted the 2014 Sustainable Transport Award (https://www.itdp.org/buenos-aires-argentina-wins-2014-sustainable-transport-award/).

### 3.4 Proxies from data on amenities

In this final section, we study the relationship between data on amenities and other types of sets of data.

#### 3.4.1 Amenities and mobility

**Fig 26:**
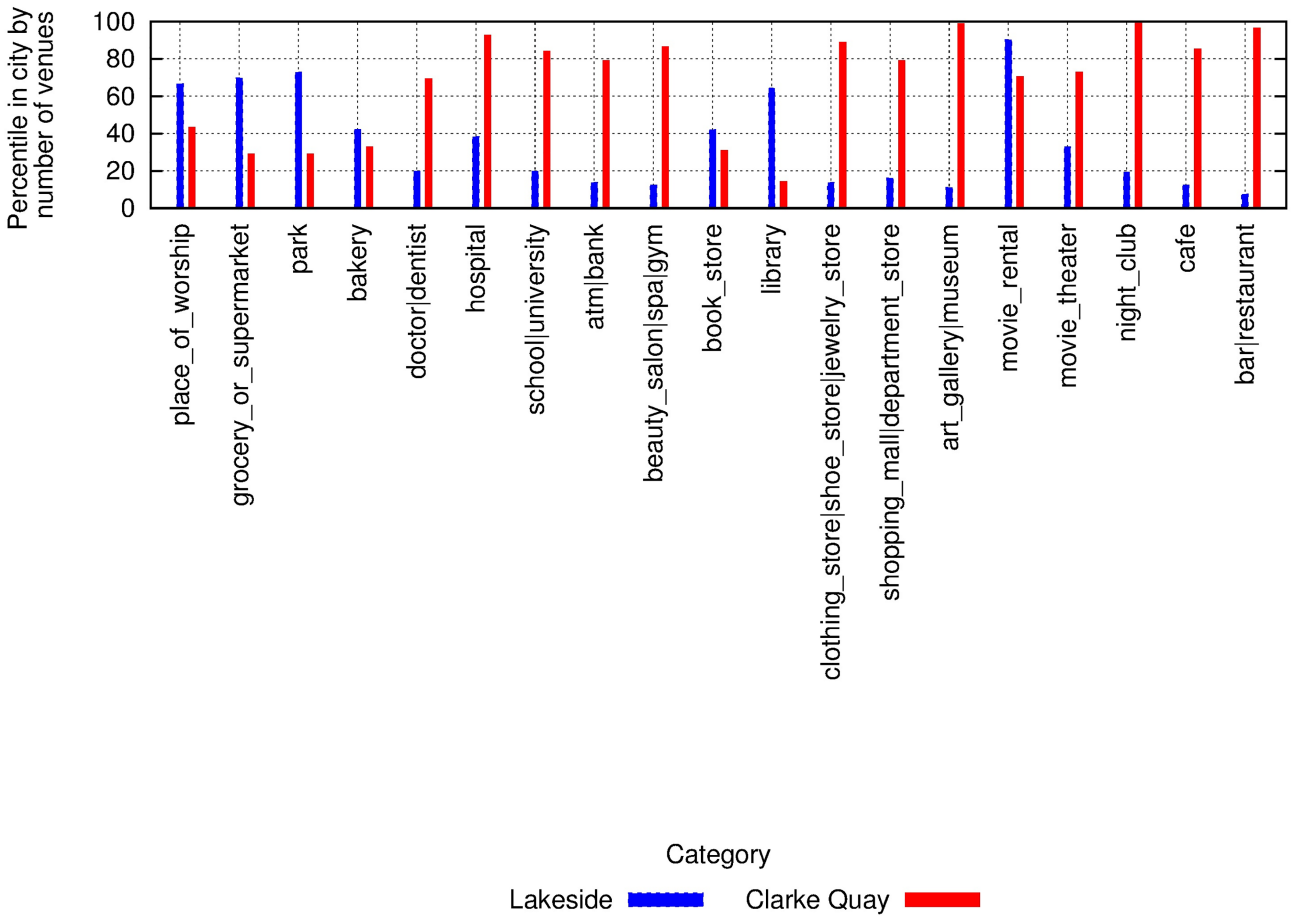
Two areas in Singapore, with their percentile ranking (y-axis) among all areas, with respect to number of venues in different categories (x-axis).

**Fig 27:**
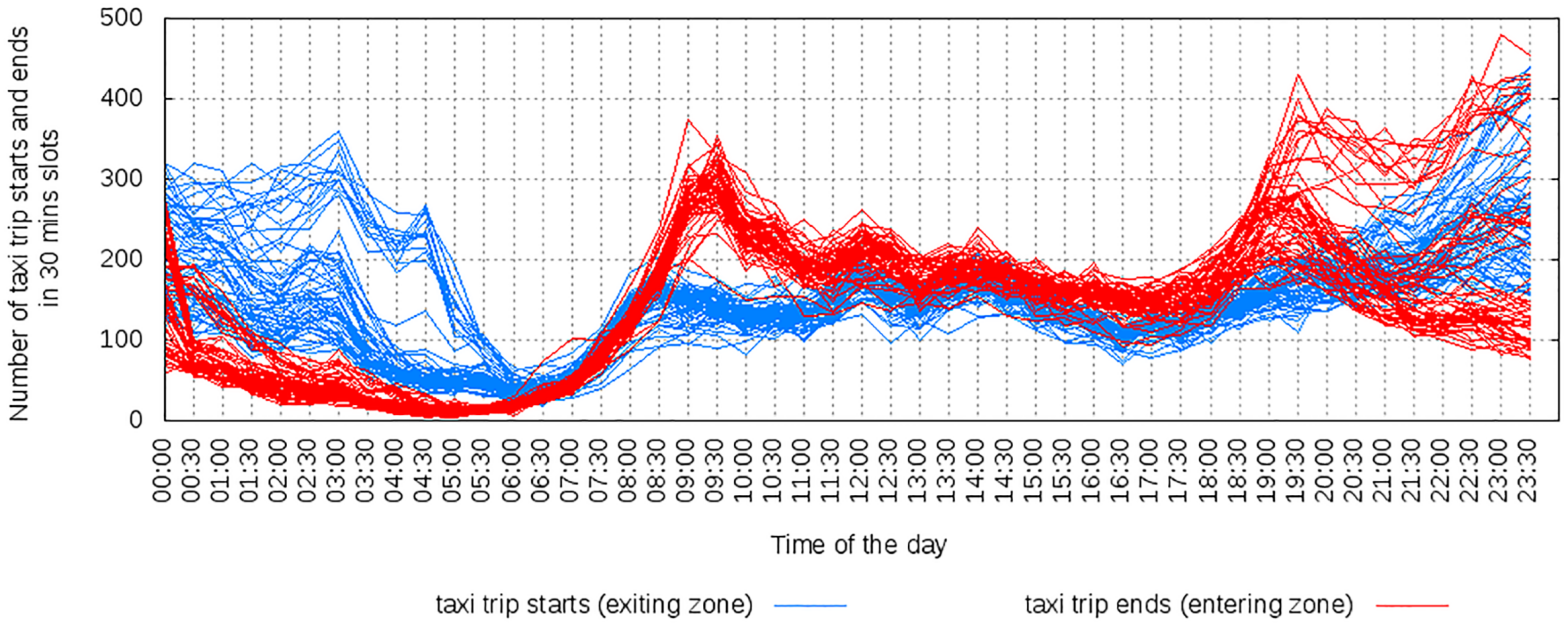
Clarke-Quay on weekdays, shows morning peak in entries and late night activities.

**Fig 28:**
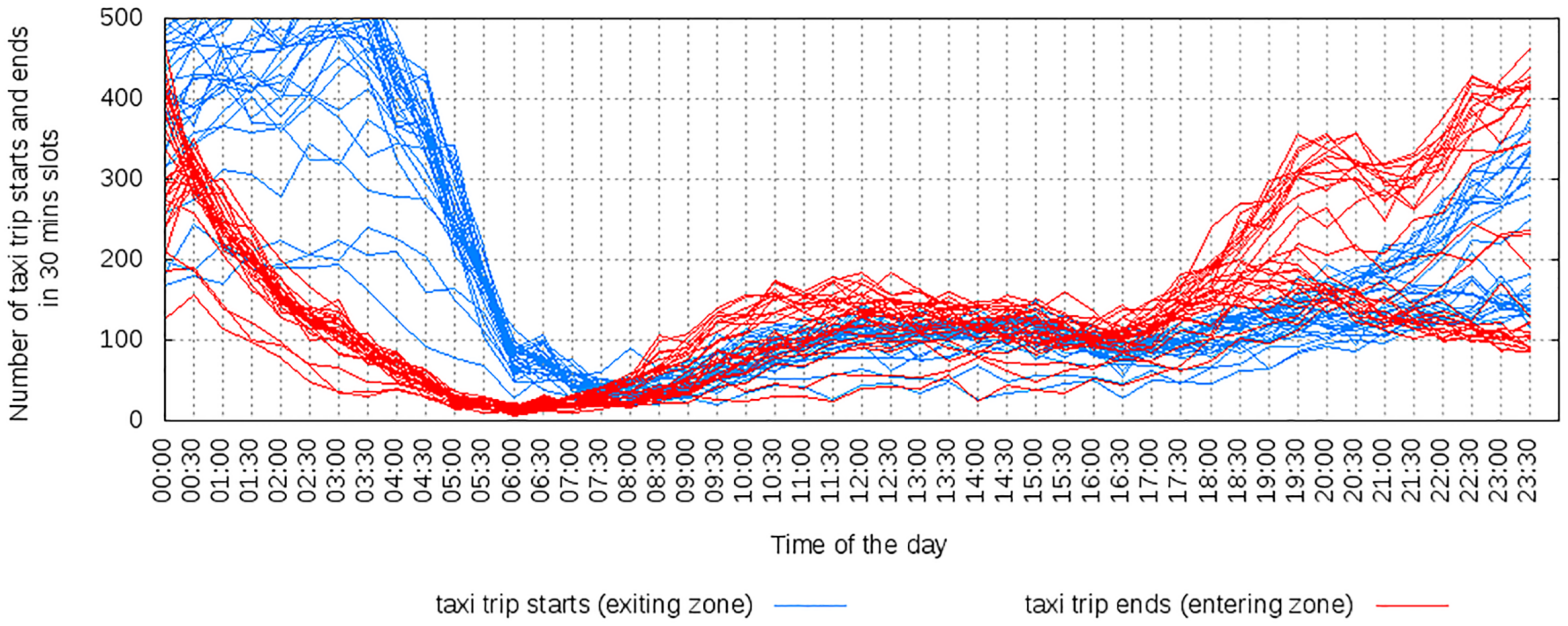
Clarke-Quay on weekends, with morning peaks suppressed and more pronounced late night activities.

**Fig 29:**
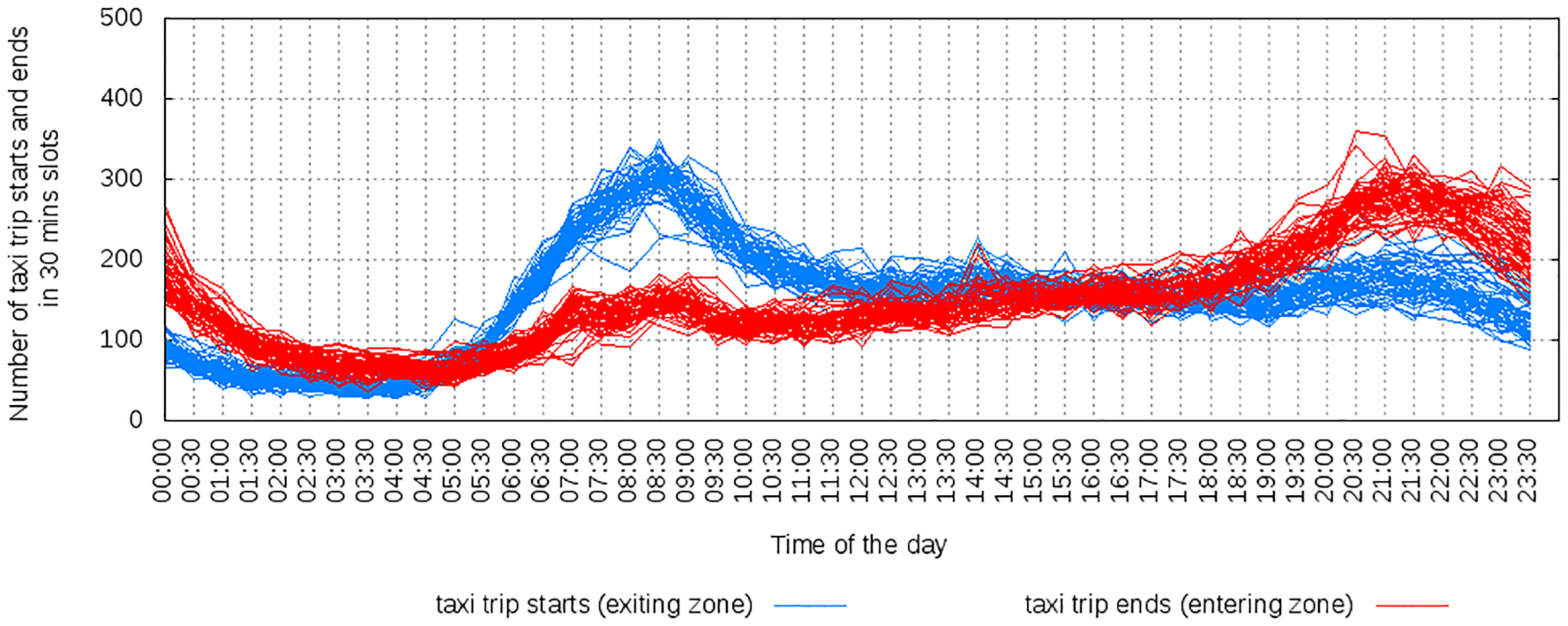
Lakeside on weekdays, with people leaving the region during morning peak hours and coming back during evening peak hours.

The amenities available in an area impact how people move in and out the area. To see how, let us consider data on amenities and taxi patterns in Singapore. For the two areas of Lakeside and Clarke Quay, each bar in Fig 26 shows one area’s percentile ranking by number of amenities in a given category. Figs 27 to 29 shows the cumulative number of trips starting and ending at the two locations over time. Lakeside is characterized by categories such as place of worship, grocery store, park, library and movie rental. As one expects, the area has two peaks, one in the morning for incoming traffic and the other in the evening for outgoing traffic. Clarke Quay, on the other hand, is characterized by night clubs, cafes, bars and restaurants, art galleries (which explain high taxi activity in the early hours of weekends), and by shopping malls, schools, universities, banks, and an hospital (which explain the peak hours in the morning on weekdays).

Mobility data is proprietary and, as such, hard to obtain. These results suggest that, in the absence of mobility data, good proxies for weekly mobility patterns could be extracted even from data on amenities.

#### 3.4.2 Amenities and street layout

Space Syntax [23] provides a graph representation of the urban layout that accounts for the way people deal with space and navigation. It simplifies the spatial geometry by reducing complex spaces into sets of points and lines [24].

The core of this methodology is the axial map: each open space (e.g., street, square) is approximated by a minimal set of straight lines (or, in technical parlance, axial lines), and their connections reflect the elements that are directly visible by humans. From the axial map, an *integration* metric can be computed. This metric counts how many turns have to be made from a street segment to reach all other street segments in the network, using shortest paths. The first intersecting segment requires only one turn, the second two turns and so on. The street segments that require the fewest turns to reach all other streets are said to be the *most integrated*.

**Fig 30:**
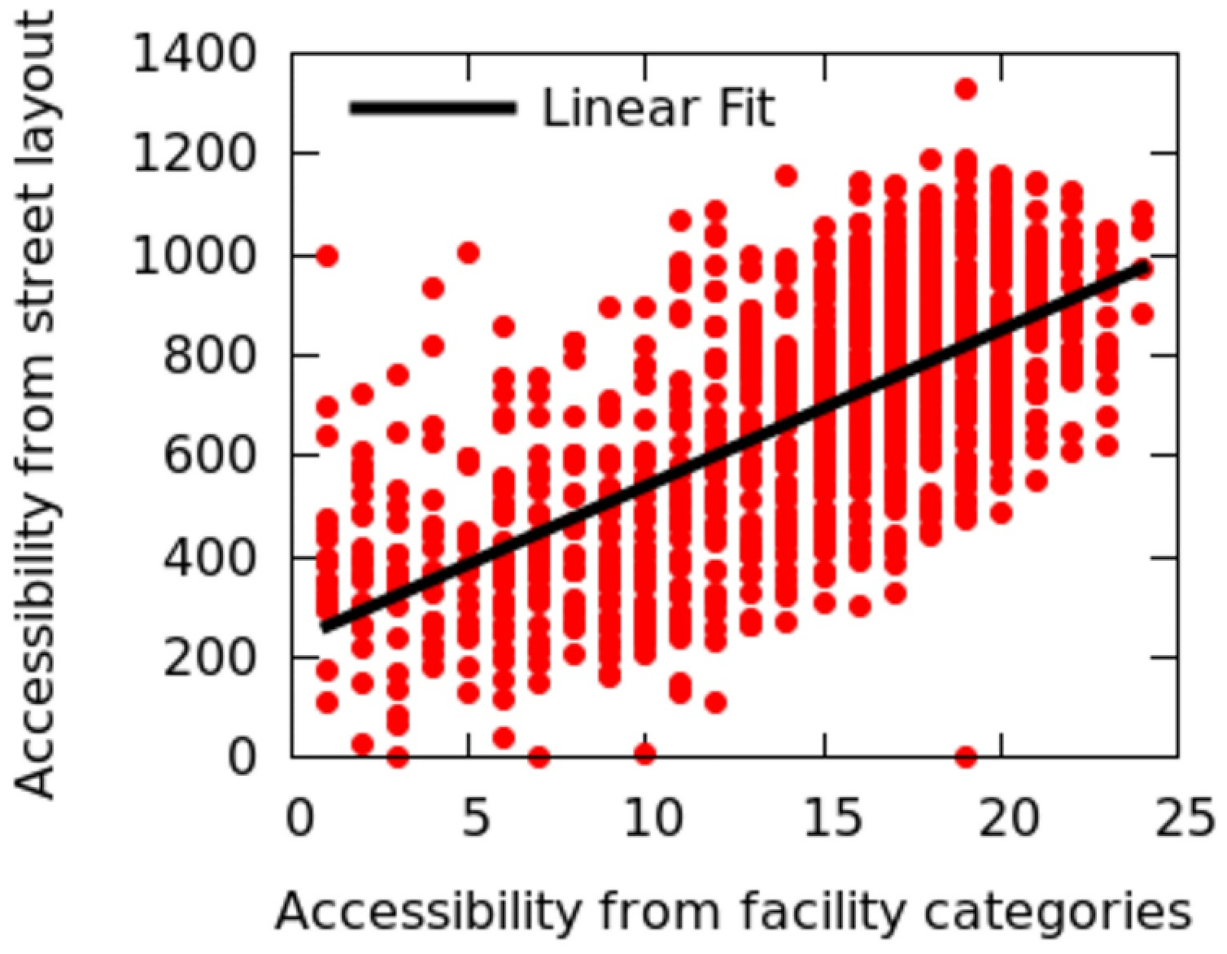
Space Syntax integration (based on the street layout) *vs*. spatial capital *C*_*a*_ (based on the availability of venues). Pearson correlation of *r* = 0.67.

We compute the integration measures for the Barcelona’s road network, which was extracted from Open Street Map. The spatial capital values *C*_*a*_ are then compared with integration values. These two metrics, computed from two distinct data sources, are correlated (Fig 30), with a Pearson Correlation coefficient of 0.67. This indicates that, the easier it is to move from one street to another (measured by the integration metric), the more accessible amenity categories happen to be (measured by the spatial capital metric). Though Space Syntax relies on open source datasets, its computation needs considerable manual processing and, as such, could be easily complemented by our simpler spatial capital metric.

## 4 Future work

In the future, researchers could explore two readily available extensions. First, they could study alternative ways of operationalizing our spatial capital metric (Eq 1). They could do so by, for example, having the number of amenities in each category within walking distance (instead of a binary number) or having a normalization by area’s population at the denominator whenever fine-grained census data is available (to account for population density).

Second, researchers could study how to set *w*_*c*_ in Eq 1, which is the importance of an amenity’s category. This weight depends on the issue under study and could be set in a variety of ways (e.g., through surveys that gather people’s preferences across multiple cities).

## 5 Related work

Leinberger *et al*. showed how an increase in access to daily errands correlates with higher house prices in neighborhoods, and with higher GDP in entire cities [25]. In their work, they also showed the importance of poly-centric urban design. Indeed, there has been considerable work on measuring the concept of poly-centricity [17, 18, 20, 21], but that work has used survey data or proprietary data (e.g., mobility traces from mobile phones, subway trips), which is costly to produce or is not always available. Often poly-centricity is associated with walkability, which again has been operationalized upon survey data, limiting the scale of services based on walkability [6, 7].

To partly counter that, Quercia *et al*. recently used data from Flickr and Foursquare to measure safety, ease of crossing, presence of sidewalks with roads in a scalable way [26]. The same authors also applied crowd-sourcing methodologies to identify urban travel routes that make people happy [27].

The work here has shown that, upon Web data, one is able not only to replicate most of the findings from those prior studies but also to provide new insights for cities that have never been studied before.

## 6 Conclusion

Using a scalable methodology, we have gathered data about amenities from the Web and put it to use for answering traditional questions in the urban planning field. We have shown how municipal authorities could inform evidence-based urban interventions, and how financial authorities could compare cities with each other (nowadays such a comparison is key to the allocation of infrastructure budget within nation states). The private sector might also benefit. Since spatial capital is associated with quality of life, websites offering, for example, house search services could readily integrate our methodology into their products.

More importantly, we have offered a methodology that allows researchers to study cities that the literature has so far neglected. To ease replicability, data gathering scripts, aggregate data about amenities, and spatial capital measurements are available on the project’s website http://goodcitylife.org/spatialcapital. Upon that data, researchers could explore how spatial capital translates into other forms of capital: does it impact house prices (financial capital) and, ultimately, the social texture of neighborhoods (social capital)?
